# Exploring the health benefits of *Ganoderma*: antimicrobial properties and mechanisms of action

**DOI:** 10.3389/fcimb.2025.1535246

**Published:** 2025-07-18

**Authors:** Samantha C. Karunarathna, Nimesha M. Patabendige, Kalani K. Hapuarachchi, Itthayakorn Promputtha

**Affiliations:** ^1^ Center for Yunnan Plateau Biological Resources Protection and Utilization, College of Biology and Food Engineering, Qujing Normal University, Qujing, Yunnan, China; ^2^ School of Medical, Molecular and Forensic Sciences, Murdoch University, Murdoch, WA, Australia; ^3^ College of Biodiversity Conservation, Southwest Forestry University, Kunming, China; ^4^ Department of Biology, Faculty of Science, Chiang Mai University, Chiang Mai, Thailand; ^5^ Environmental Science Research Center (ESRC), Chiang Mai University, Chiang Mai, Thailand; ^6^ Natural Extracts and Innovative Products for Alternative Healthcare Research Group, Faculty of Science, Chiang Mai University, Chiang Mai, Thailand

**Keywords:** antimicrobial activity, biofilm inhibition, pathogenic bacteria, polysaccharides, synergistic effects, triterpenoids

## Abstract

*Ganoderma*, a well-known medicinal mushroom, has garnered attention for its broad therapeutic properties, particularly its potent antimicrobial activities. This review focuses on the mechanisms of action and bioactive compounds responsible for the ability of *Ganoderma* to inhibit various pathogenic microorganisms. The polysaccharides, triterpenoids, proteins, and phenolic compounds in *Ganoderma* exhibit strong antimicrobial effects by targeting bacterial cell walls, disrupting membrane integrity, and inhibiting key microbial enzymes. These compounds are effective against a wide range of bacteria, including *Staphylococcus aureus*, *Escherichia coli*, *Pseudomonas aeruginosa*, and various fungi. Triterpenoids, specifically, have demonstrated efficacy in modulating immune responses, further enhancing the body’s defense mechanisms against infections. Furthermore, the role of *Ganoderma* in preventing biofilm formation and combating antibiotic-resistant strains highlights its potential as a natural antimicrobial agent. While *in vitro* and *in vivo* studies strongly support the antimicrobial properties of *Ganoderma*, future resety -50arch should focus on large-scale clinical trials to confirm its efficacy and explore its synergistic effects with conventional antibiotics. Establishing standardized dosages and exploring the molecular pathways of its antimicrobial actions will be key to incorporating *Ganoderma* into clinical practice for infection control.

## 1 Introduction

*Ganoderma* is a genus of medicinal mushrooms used for thousands of years in traditional East Asian medicine. Revered for its numerous therapeutic benefits, *Ganoderma* has gained significant attention in modern scientific research due to its bioactive compounds exhibiting various pharmacological activities (Karunarathna et al., 2024a). Among these activities, its antimicrobial properties stand out as an area of growing interest, particularly in an era where antimicrobial resistance (AMR) poses a significant global health threat (Pandey et al., 2020). Understanding the mechanisms by which *Ganoderma* exerts its antimicrobial effects is critical for developing novel therapies that harness its bioactive compounds to combat various infectious diseases (Mousavi et al., 2023; Karunarathna et al., 2024b). The antimicrobial properties of *Ganoderma* are attributed primarily to its rich content of bioactive compounds such as polysaccharides, triterpenoids, phenolic compounds, proteins, and peptides (Ahmad, 2019; Cadar et al., 2023). These compounds have been shown to work synergistically to inhibit the growth of various pathogenic microorganisms, including bacteria, fungi, and viruses. Historically, *Ganoderma* has been used in traditional medicine to treat infections, improve immune function, and promote overall health. These traditional uses are being validated by scientific research, which has provided evidence for *Ganoderma*’s effectiveness in inhibiting microbial growth and enhancing immune responses to infections.

One of the most studied bioactive compounds in *Ganoderma* is polysaccharides, particularly β-glucans, which are known to modulate immune responses and exhibit strong antimicrobial effects. Polysaccharides have been shown to activate macrophages and other immune cells, enhancing the ability of the body to detect and eliminate microbial pathogens. Triterpenoids, another significant class of compounds in *Ganoderma*, have demonstrated the ability to disrupt microbial cell walls and inhibit the replication of pathogens, particularly bacteria and fungi (Liu et al., 2022). In addition to these, phenolic compounds and polyketides of farnesyl quonines types and peptides isolated from *Ganoderma* also play crucial roles in its antimicrobial activity by scavenging free radicals, reducing oxidative stress, and enhancing the body’s natural defense mechanisms (Basnet et al., 2017). The antimicrobial properties of *Ganoderma* have been documented in various *in vitro* and *in vivo* studies, which have explored its efficacy against a wide range of pathogens. For instance, *Ganoderma* has potent inhibitory effects on Gram-positive and Gram-negative bacteria, including *Staphylococcus aureus*, *Escherichia coli*, and *Pseudomonas aeruginosa*. Moreover, it has shown antifungal activity against *Candida albicans*, a common cause of fungal infections in immunocompromised individuals (Ahmad et al., 2024). Furthermore, emerging studies have investigated its potential antiviral activity, with some evidence suggesting that *Ganoderma* extracts may inhibit the replication of viruses such as herpes simplex virus (HSV) and influenza virus (Seo and Choi, 2021). These findings suggest that *Ganoderma* could be a valuable natural alternative or adjunct to conventional antimicrobial therapies, particularly in the context of rising antibiotic resistance. The mechanisms through which *Ganoderma* exerts its antimicrobial effects are complex and multifaceted. Disruption of microbial cell walls, inhibition of nucleic acid synthesis, and modulation of immune responses are among the primary mechanisms identified in current research. *Ganoderma* bioactive compounds interact with microbial cells, weakening their structural integrity and preventing proliferation. Moreover, *Ganoderma*’s ability to modulate the host’s immune system enhances its antimicrobial efficacy, as it not only directly inhibits pathogens but also strengthens the body’s natural defenses against infections (Gao et al., 2005). Despite the promising antimicrobial potential of *Ganoderma*, several challenges remain. One major limitation is the variability in the composition of bioactive compounds across different *Ganoderma* species and even within the same species depending on environmental factors and cultivation methods. This variability makes it difficult to standardize extracts for clinical use. In addition, while *in vitro* and animal studies have provided valuable insights, more human clinical trials are needed to confirm the safety and efficacy of *Ganoderma* as an antimicrobial agent. Future research should focus on identifying the compounds responsible for antimicrobial effects of *Ganoderma* and developing standardized formulations for therapeutic use. *Ganoderma* represents a promising natural source of antimicrobial agents with potential applications in treating various infections. Its ability to modulate the immune system and directly inhibit microbial growth makes it an attractive candidate for developing novel antimicrobial therapies. However, further research is necessary to fully understand its mechanisms of action and overcome the challenges associated with its variability and standardization. As antibiotic resistance continues to rise globally, exploring natural alternatives such as *Ganoderma* is becoming increasingly important. This review aims to provide a comprehensive overview of the antimicrobial properties of *Ganoderma*, focusing on recent advances in understanding its bioactive compounds, mechanisms of action, and potential therapeutic applications, particularly in the context of rising AMR. The novelty of this work lies in synthesizing recent findings and highlighting emerging insights into the role of *Ganoderma* as a promising natural antimicrobial agent.

## 2 *Ganoderma* bioactive compounds

*Ganoderma* species produce a variety of bioactive compounds with significant health benefits, including polysaccharides, triterpenoids, proteins, peptides, and phenolic compounds, each contributing uniquely to their therapeutic potential. This section provides a brief overview of these compounds, highlighting their structures, functions, and mechanisms of action. Detailed phytochemical and bioactivity profiles of *Ganoderma* have been extensively reviewed (Baby et al., 2015; Blundell et al., 2023).

Among the most studied bioactive compounds are the polysaccharides, particularly β-glucans from *G. lucidum*. These complex carbohydrates, characterized by β-D-glucose linkages, are categorized by molecular weight and solubility, factors that influence their biological activities (Karunarathna et al., 2024a). β-glucans are known to modulate the immune system by activating macrophages and natural killer cells, enhancing the immune response of the host (Chen et al., 2023). They also impact cellular signaling pathways, regulating cytokine production and inhibiting tumor growth (Zhang et al., 2023). The structural features of *Ganoderma* polysaccharides, such as branching patterns and molecular configurations, play a critical role in determining their therapeutic efficacy (Wu et al., 2025).

Triterpenoids, another major class of *Ganoderma* bioactive compounds, include ganoderic and lucidenic acids. These compounds, with their multi-ring structures and diverse functional groups, contribute to a wide range of biological activities (Raza et al., 2024; Pan et al., 2025). Triterpenoids have shown potent immunomodulatory effects by modulating cytokine production and enhancing the activity of immune cells like T cells and macrophages (Jin et al., 2025; Lucius, 2025). They also demonstrate broad-spectrum antimicrobial activity by disrupting microbial cell membranes and interfering with enzymatic processes critical for pathogen survival (Ahmad et al., 2024; Wang et al., 2017; Ewunkem et al., 2024; Liang et al., 2024). Phenolic compounds in *Ganoderma*, such as flavonoids, phenolic acids, and polyphenols, are well-known for their antioxidant properties. They reduce oxidative stress by neutralizing free radicals and reactive oxygen species (ROS). Their antioxidant effects are largely due to their electron-donating ability, stabilizing free radicals and preventing cellular damage and inflammation (Kebaili et al., 2021; Plosca et al., 2025). In addition to their antioxidative functions, phenolic compounds also exert antimicrobial activity by disrupting microbial cell structures and inhibiting key enzymatic functions necessary for pathogen survival (Rašeta et al., 2023). The multifunctional roles of these compounds underscore their significance in maintaining health and preventing disease.

## 3 Mechanisms of antimicrobial action

**Figure 1:**
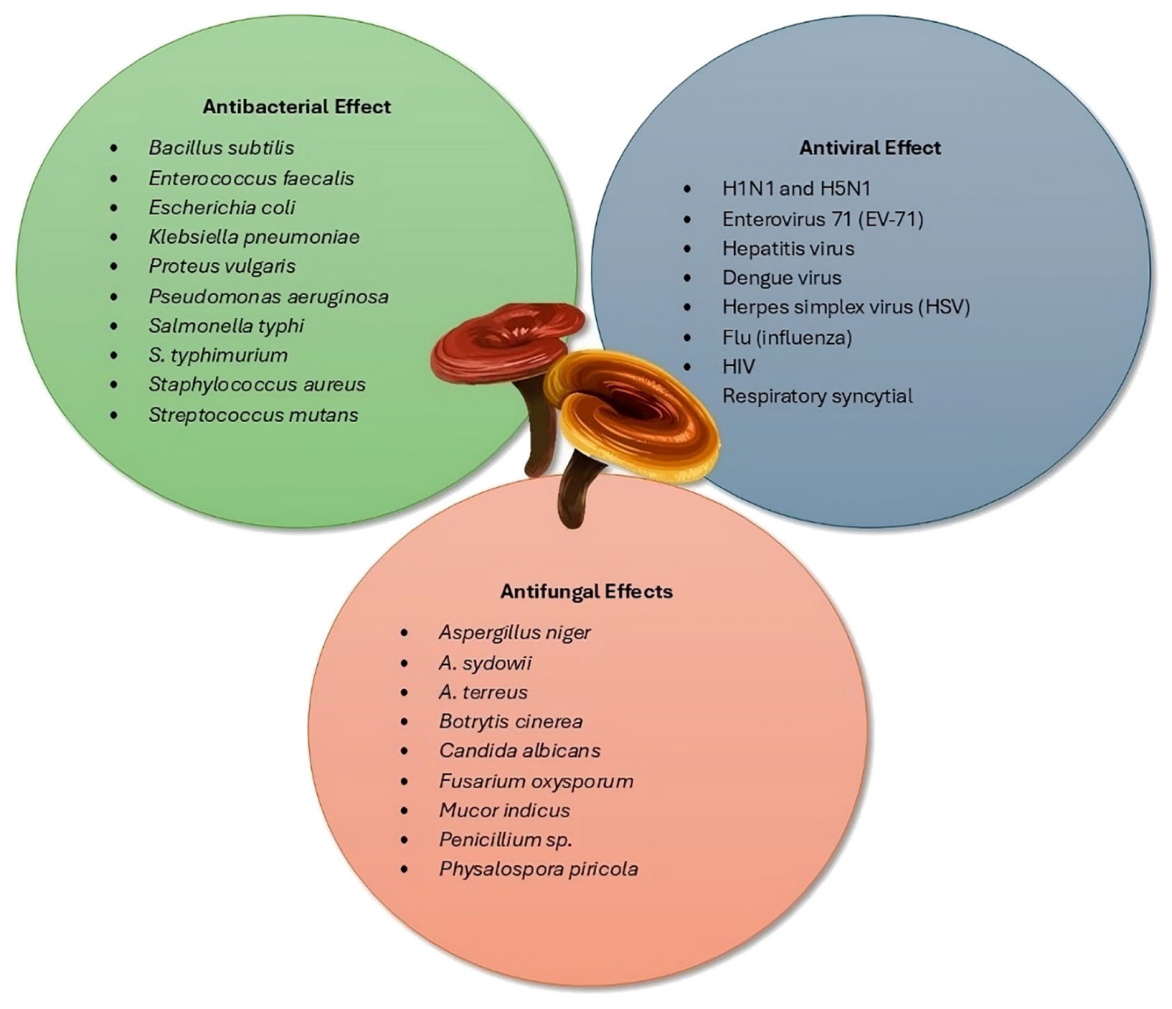
Potential antimicrobial properties of *Ganoderma* (Ahmad et al., 2024).

*Ganoderma* species possess many bioactive compounds that exhibit significant antimicrobial activities. The mechanisms by which these compounds act against pathogens are multifaceted, involving direct effects on microbial structures and functions and modulation of the host immune system (**Figure 1**).

### 3.1 Disruption of microbial cell walls

One of the primary antimicrobial mechanisms of *Ganoderma* bioactive compounds is the disruption of microbial cell walls. Triterpenoids, such as ganoderic acids found in *Ganoderma lucidum*, interact with the lipid components of bacterial and fungal cell membranes, leading to increased permeability and cell lysis. This disruption compromises the integrity of the microbial cell wall, causing leakage of cellular contents and, ultimately, cell death (Ewunkem et al., 2024; Ojha, 2025).

### 3.2 Inhibition of nucleic acid synthesis

*Ganoderma* bioactive compounds also inhibit microbial proliferation by interfering with nucleic acid synthesis. Polysaccharides extracted from *Ganoderma* species have been reported to inhibit DNA and RNA synthesis in pathogenic microbes. They achieve this by binding to nucleic acids or key enzymes involved in replication and transcription processes, thereby hindering microbial growth and replication. This inhibition of genetic material synthesis is crucial in preventing the spread and survival of the pathogen (Sułkowska-Ziaja et al., 2022; Liang et al., 2024).

### 3.3 Immune modulation

*Ganoderma* compounds enhance the immune response of the body, providing an indirect mechanism to combat infections. Polysaccharides, especially beta-glucans, are known to modulate the immune system by activating macrophages, dendritic cells, and natural killer cells (Zhang et al., 2023; Zhong et al., 2024). This activation increases cytokine and antibody production, bolstering the body’s ability to fight microbial invaders. The immunomodulatory effects of *Ganoderma* not only enhance the innate immune response but also promote adaptive immunity. By stimulating immune cell proliferation and differentiation, these compounds help establish long-term immunity against specific pathogens (Seweryn et al., 2021; Zhong et al., 2023). This dual action makes *Ganoderma* an effective agent in preventing and managing infections.

### 3.4 Oxidative stress regulation

Oxidative stress plays a significant role in the pathogenesis of many microbial infections. Phenolic compounds of *Ganoderma* exhibit strong antioxidant properties, which help balance ROS within microbial cells (Zahmoul et al., 2024; Plosca et al., 2025). By inducing oxidative stress beyond the tolerance levels of microbes, these compounds can lead to cellular damage and death of the pathogens. Conversely, in host cells, *Ganoderma* antioxidants protect against oxidative damage caused by infections. They scavenge excess ROS, reducing inflammation and preventing tissue damage (Ahmad et al., 2024; Chen et al., 2024). This protective effect supports the healing process and restores normal cellular functions.

## 4 Synergistic effects of compounds

The antimicrobial efficacy of *Ganoderma* species, particularly *G. lucidum*, is not solely attributed to individual bioactive compounds. Instead, the interactions between various compounds—such as polysaccharides, triterpenoids, proteins, peptides, and phenolic compounds—create synergistic effects that significantly enhance their therapeutic potential. Synergy refers to the increased effectiveness when these compounds work together, often producing results greater than the sum of their actions.

### 4.1 Interaction between different bioactive compounds

Polysaccharides and triterpenoids are two of the most studied bioactive compounds in *Ganoderma*. Polysaccharides are known for their immunomodulatory properties, while triterpenoids have potent antimicrobial and anti-inflammatory activities. Combined, these two compounds demonstrate enhanced immunomodulatory effects, stimulating the body’s immune system to fight off infections more effectively (Gao et al., 2005). For example, while triterpenoids may directly disrupt microbial cell membranes, polysaccharides boost the production of immune cells like macrophages and natural killer (NK) cells, leading to a synergistic antimicrobial action (Seweryn et al., 2021; Zhong et al., 2023).

#### 4.1.1 Proteins and peptides with triterpenoids

Proteins and peptides in *Ganoderma* also exhibit antimicrobial properties, particularly against bacteria and fungi. When these are used with triterpenoids, the compounds together demonstrate enhanced efficacy. The peptides may disrupt microbial membranes, while triterpenoids inhibit nucleic acid synthesis, thereby preventing microbial replication. This dual mechanism increases the effectiveness of the antimicrobial response, especially in pathogens resistant to single-compound treatments (Cör Andrejč et al., 2022; Cadar et al., 2023).

#### 4.1.2 Phenolic compounds and polysaccharides

Phenolic compounds in *Ganoderma* contribute significantly to its antioxidant activity, reducing oxidative stress within cells. When combined with polysaccharides, these phenolic compounds enhance the immune response and improve the organism’s overall resistance to microbial infections. The phenolic compounds neutralize ROS, while polysaccharides improve immune cell signaling. This combination leads to a more efficient and sustained immune response to pathogens, particularly in cases of chronic infections (Seweryn et al., 2021; Chen et al., 2024).

### 4.2 Enhanced antimicrobial activity

Research shows that combining polysaccharides and triterpenoids from *Ganoderma* results in enhanced antibacterial activity. For example, studies on *E. coli* and *S. aureus* have shown that combining these two compounds leads to stronger inhibition of bacterial growth compared to their individual effects. The synergy is observed in the disruption of bacterial cell walls by triterpenoids and the enhancement of immune responses by polysaccharides, which work together to eliminate bacterial infections more efficiently (Ahmad et al., 2024).

#### 4.2.1 Synergistic effects against fungal infections

In the case of fungal infections, particularly *C. albicans*, combining polysaccharides with phenolic compounds has been shown to enhance antifungal activity. This combination disrupts fungal cell walls while simultaneously inducing oxidative stress within the fungal cells. The phenolic compounds reduce ROS accumulation, which damages fungal cells, and the polysaccharides enhance the immune response, creating a powerful antifungal effect. The result is a more effective inhibition of *C. albicans* growth and biofilm formation, critical for fungal survival and virulence (Roychoudhury et al., 2024).

#### 4.2.2 Viral infections

Emerging research also suggests that the synergistic effects of polysaccharides and triterpenoids in *Ganoderma* may extend to viral infections. For instance, in studies on the HSV, a combination of these compounds has demonstrated the ability to inhibit viral replication more effectively than when either compound is used alone. Polysaccharides stimulate immune responses, such as activating macrophages and NK cells, while triterpenoids interfere with viral entry into host cells, resulting in enhanced antiviral activity (Eo et al., 2000; Bharadwaj et al., 2019).

## 5 Research on specific microorganisms

*Ganoderma* species, particularly *G. lucidum*, have gained recognition for their potent antimicrobial properties against various pathogens. The bioactive compounds in *Ganoderma* exhibit broad-spectrum activity against bacteria, fungi, and viruses, making it a promising natural remedy in combating infections. Below is a detailed review of research focusing on the effects of *Ganoderma* on specific microorganisms. GTs are the most common antimicrobial and antiparasitic compounds reported from *Ganoderma* sp. Farnesyl quinone, a polyketide type, is the second most common antimicrobial and antiparasitic compound from *Ganoderma* sp. Quinones are known to be oxidized derivatives of aromatic compounds and are often readily made from reactive aromatic compounds with electron-donating substituents such as catechols and phenols. Besides GTs, polypeptides, small peptides such as ganodermin, polysaccharides such as sacchachitin, and chitosan also possess antimicrobial and antiparasitic properties (Mothana et al., 2000; Wang and Ng, 2006; Sanodiya et al., 2009; Chuang et al., 2013). Extracts from fruiting bodies, both wild and cultivated, and mycelia from fermentation broth are used for the isolation of antimicrobial and antiparasitic bioactive compounds. Literature divulges that, most commonly, ethanol (EtoAc) is used to prepare crude extract; sometimes, some researchers prefer other solvents such as chloroform (CHCl_3_), EtOH, and acetone (Isaka et al., 2016). In addition, our review reveals that hexane and ether are poorly used for the preparation of extract from *Ganoderma* sp. Moreover, some techniques such as microwave, ultrasound, and enzyme treatments can facilitate the breakdown of the cell wall (Ferreira et al., 2015). Solvents like MeOH, EtOH, CH_2_Cl_2_, CHCl_3_, and aqueous—both cold and hot—are used for further purification and isolation. Techniques such as thin-layer chromatography (TLC), high-performance liquid chromatography (HPLC), and column chromatography (CC) are used to facilitate the purification and isolation process (Huie and Di, 2004).

### 5.1 Bacterial infections

**Figure 2:**
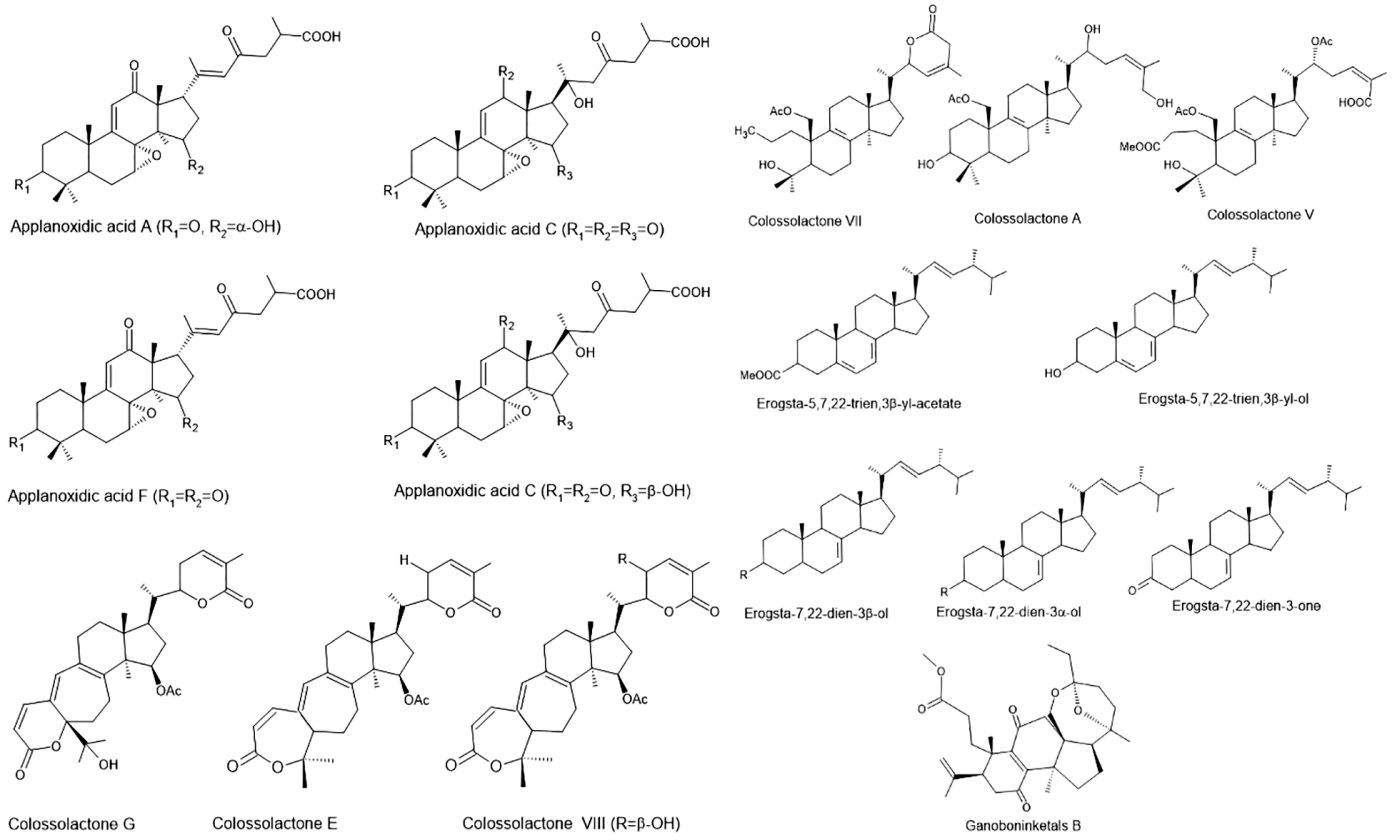
Structures of bioactive compounds from *Ganoderma* species with antimicrobial and antiparasitic effects (Basnet et al., 2017).

Several studies have demonstrated the efficacy of *Ganoderma* bioactive compounds against pathogenic bacteria, including both Gram-positive and Gram-negative strains. Key compounds, such as triterpenoids, polysaccharides, and peptides, have shown significant antibacterial effects (**Figure 2**). *Ganoderma* has been reported as an important source of antimicrobial bioactive compounds. Terpenes, terpenoids, and polyketides of farnesyl quonine types are the major secondary metabolites produced by *Ganoderma* sp. In *Ganoderma* species, more than 316 terpenes have been reported, with the majority of compounds from *G. lucidum* (Xia et al., 2014).

#### 5.1.1 *Ganoderma* extracts and fermentation broths

The methanol extract of *G. lucidum* showed antibacterial activity against *E. coli*, *Salmonella typhimurium*, and *Bacillus subtilis* [minimum inhibitory concentration (MIC): 1 mg/well], with bioactive polyphenols, flavonoids, quinones, and terpenes identified (Sheena et al., 2003). Among 23 Yemeni Basidiomycetes, *Agaricus* sp., *Coriolopsis caperata*, *Ganoderma colossus*, *Ganoderma resinaceum*, *Phellorinia herculea*, and *Tulostoma obesum* exhibited potent antibacterial effects, while *G. resinaceum*, *Inonotus ochroporus*, *Phellinus rimosus*, and *P. herculea* displayed strong antioxidant activity (Al-Fatimi et al., 2005). *G. lucidum* butanol extracts inhibited microbial growth and disrupted fungal spore formation, suggesting potential for antimicrobial tea formulations (Rofuli et al., 2005). *Ganoderma applanatum*, *Tricholoma crassum*, and *Trametes corrugata* showed peak antibacterial activity (terpenoids and polysaccharides) after 16 days of fermentation (Bhattacharyya et al., 2006). *Ganoderma* spp. (e.g., *G. carnosum*) exhibited static, heat-stable effects against pathogens like *E. coli* and *C. albicans* (Yamac and Bilgili, 2006). Furthermore, chitosan from *Ganoderma tsugae* outperformed doxycycline against *Actinobacillus actinomycetemcomitans*, retaining 56.58% activity after 18 days, highlighting dental applications (Chen et al., 2007). *G. lucidum* aqueous extracts (from *Persia americana* logs) showed stronger antibacterial effects than methanol extracts (Ofodile and Bikomo, 2008), while its chloroform extracts inhibited Gram-positive and Gram-negative bacteria via sterols and triterpenoid acids (Keypour et al., 2008). In addition, *G. applanatum* methanol extracts (rich in palmitic acid) selectively inhibited Gram-negative bacteria (Moradali et al., 2008).

*G. applanatum* exhibited antimicrobial activity against *E. coli*, *S. aureus*, *C. albicans*, *Mycobacterium smegmatis*, and *Sporothrix schenckii*, highlighting its therapeutic potential (Barranco et al., 2010). *G. lucidum* methanol, ethanol, and aqueous extracts showed potent activity against pathogens like *Listeria monocytogenes* and methicillin-resistant *S. aureus* (MRSA), with methanol being the most effective solvent (Aneeshia and Sornaraj, 2010). *G. applanatum* methanol extract displayed strong DPPH scavenging (82.80%), while *G. lucidum* chloroform extract had notable antioxidant and antibacterial effects, linked to high phenol content (Karaman et al., 2010). *G. lucidum* inhibited spore germination of *Alternaria brassicicola*, suggesting its potential as a biocontrol agent (Chen and Huang, 2010). Methanol, acetone, chloroform, and aqueous extracts of *G. lucidum* mycelia inhibited Gram-positive and Gram-negative bacteria (100 mg/mL), with Gram-positive strains more susceptible (Kamble et al., 2011). Furthermore, in Pakistan, Lahore isolates of *G. lucidum* (G-1, G-3, and G-5) inhibited *Xanthomonas* spp., while G-2 and G-4 were effective against *E. coli* and *Pseudomonas* spp., respectively (Nasim and Ali, 2011). *G. lucidum* aqueous extract (200 mg) showed a 31-mm inhibition zone against *S. typhi* and *S. aureus*, while its methanolic extract was most antifungal (30 mm against *Mucor indicus*) (Sekaran et al., 2011). Ethyl acetate extracts of *Ganoderma praelongum* sesquiterpenoids were highly active against MRSA (MIC: 0.390–6.25 mg/mL), unlike ineffective polysaccharides (Ameri et al., 2011). *Ganoderma carnosum* dichloromethane extracts strongly inhibited *S. aureus* and *Micrococcus luteus* (Srivastava and Sharma, 2011). *Ganoderma formosanum* polysaccharides (d-mannose, d-galactose, and d-glucose) enhanced macrophage activity (TNF-α, nitric oxide, and phagocytosis) and protected mice against *L. monocytogenes* (Wang et al., 2011).

*G. lucidum* ethyl acetate extracts showed the strongest antibacterial activity (containing carbohydrates, saponins, and terpenoids), being most effective against *Corynebacterium pyogenes*, *B. subtilis*, and *Klebsiella pneumoniae* though less potent than Ampiclox^R^ (Shamaki et al., 2012). Water extracts inhibited *P. aeruginosa*, *Proteus vulgaris*, and *Enterococcus faecalis* but not *L. monocytogenes*, while hexane/dichloromethane/ethyl acetate showed limited antimicrobial isolation potential (Kamra and Bhatt, 2012). In Central India, *G. lucidum* aqueous extracts enhanced synthetic antibiotics against *S. aureus*, *K. pneumoniae*, *Bacillus cereus*, and *P. aeruginosa* (Karwa and Rai, 2012). Acetone extracts showed the strongest activity against *P. aeruginosa* (33 mm zone) and the weakest against *S. aureus/K. pneumoniae* (7 mm), with MICs of 4–35 mg/mL (Mehta and Jandaik, 2012). *G. lucidum* spore and *G. applanatum* polysaccharides inhibited *S. aureus*, *B. cereus*, and *Salmonella enteritidis*, suggesting potential as food supplements (Klaus et al., 2012). Comparative studies showed that *G. lucidum* had the largest inhibition zones against *E. coli/Klebsiella* sp., though less than standard antibiotics (Krishnaveni and Manikandan, 2014). Solvent choice significantly impacted activity: benzene extracts best inhibited *E. coli/Neisseria meningitidis* (Shikongo, 2012), while methanol extracts surpassed ampicillin/streptomycin against *S. aureus/B. cereus* (MIC: 0.0125–0.75 mg/mL) (Heleno et al., 2013). Diethyl ether/chloroform extracts showed strong antagonistic effects (Nithya et al., 2013). Traditional Namibian uses were validated as *Ganoderma* spp. showed potent Gram-positive/negative activity (Shikongo et al., 2013). The anti-*S. aureus* activity of *G. applanatum* was linked to soluble saponins/phenols (Nagaraj et al., 2013). *G. lucidum*, *Pleurotus* spp., and *Agaricus bisporus* demonstrated broad therapeutic potential (Mondal, 2013).

*G. lucidum* extracts showed significant antimicrobial activity against *P. aeruginosa*, *E. coli*, *S. aureus*, *Proteus mirabilis*, and *K. pneumoniae*. Aqueous extracts produced 11.0- to 14.0-mm inhibition zones, with bioactive tannins, phenolics, flavonoids, and saponins identified (Fakoya et al., 2013). HPTLC analysis revealed six flavonoids and four phenolics, with methanol extracts most effective against *K. pneumoniae* (24 ± 0.666 mm), while Gram-negative bacteria showed greater susceptibility than Gram-positive *S. aureus* (Sakthivigneswari and Dharmaraj, 2013). *G. praelongum* (0.3%) combined with *Glycyrrhiza glabra* (2.5%) in topical gels significantly inhibited MRSA and enhanced wound healing (Ameri et al., 2013). *G. tsugae* methanol extracts were most active against *E. coli* (20 ± 0.577 mm), with Gram-negatives more susceptible than Gram-positives (Ganesan and Dharmaraj, 2013). *G. applanatum* showed particular efficacy against Gram-positive bacteria (Pushpa et al., 2013). *G. lucidum* ethanol extracts inhibited *Helicobacter pylori* (MIC < 3 mg/mL) and *S. aureus* (MIC 10 mg/mL) but not *E. coli* (Shang et al., 2013). *G. lucidum* methanol extracts were active against *E. coli*, *S. aureus*, *B. cereus*, *Enterobacter aerogenes*, and *P. aeruginosa* (Alves et al., 2013).

Recent studies have demonstrated significant antimicrobial potential in various *Ganoderma* species. *Ganoderma boninense* methanol extracts exhibited strong activity against foodborne pathogens *E. coli* and *S. aureus*, with GC-MS analysis identifying dodecanoic acid and octadecanoic acid as key bioactive compounds (Ismail et al., 2014). Comparative research on *G. lucidum* strains revealed distinct bioactive profiles, with Serbian specimens showing higher sugar content and anticancer properties, while Chinese varieties contained more organic acids and demonstrated superior antioxidant capacity—both strains displayed antimicrobial effects that occasionally surpassed standard drugs (Stojković et al., 2014). The extraction method significantly influenced activity, as *G. lucidum* methanolic extracts (500 µg/disc) produced the largest inhibition zones (13.04 mm) against *S. aureus* and *P. aeruginosa* (Djide et al., 2014). Chloroform extracts showed notable efficacy against *S. typhi* (18 mm) and *C. albicans* (17 mm), with analytical techniques confirming triterpenoids and polysaccharides as active components (Gowrie et al., 2014). Optimized fermentation protocols yielded extracts with antioxidant activity exceeding ascorbic acid and antimicrobial effects against *Shigella dysenteriae*, *E. faecalis*, and *K. pneumoniae* (Paliya et al., 2014). Additional studies confirmed variable but promising activity of *G. lucidum* against *P. aeruginosa*, *E. coli*, *E. faecalis*, *S. aureus*, and *C. albicans*, with ethanol and chloroform extracts proving most effective (Avcı et al., 2014).

Comparative studies of mushroom species revealed that *G. tsugae* had the highest dry weight (16.1 g/100 g), while *A. bisporus* contained superior protein (32.0 mg/g) and glucose (13.2 mg/g) content. *A. bisporus* acetone extracts showed antimicrobial activity against *E. coli* (13 mm) and *P. aeruginosa* (14 mm), whereas *G. tsugae* displayed stronger antibacterial effects in DMSO extracts (Dharmaraj et al., 2014). Nigerian studies of *G. lucidum* ethanolic extracts identified steroids, triterpenoids, and glycosides with activity against *E. coli* (12 mm), *K. pneumoniae* (12 mm), *P. mirabilis* (13 mm), and *Streptococcus* spp. (14 mm) at 1,000 mg/mL (Etim et al., 2014). *Ganoderma* sp. DKR1 contained saponins, tannins, and terpenoids, with ethyl acetate extracts active against *Micrococcus* sp., *S. aureus*, and *Salmonella* sp., while chloroform extracts inhibited *E. faecalis* and *Candida* sp (Rajesh and Dhanasekaran, 2014). *G. lucidum* acetone extracts (50 µg/mL) showed potent antibacterial activity (31.60 ± 0.10 mm) against six bacterial species and antifungal effects at 1,000 mg/mL (Singh et al., 2014). With rising drug resistance, *G. lucidum* methanolic extracts containing carbohydrates, triterpenoids, and phenolics demonstrated strong antibacterial effects (Shah et al., 2014). *G. lucidum* spore powder inhibited *Prevotella intermedia* (MIC 3.62 mcg/mL) in 65% of periodontal samples (Nayak et al., 2015). *Ganoderma australe* exhibited antimicrobial and antioxidant activity from alkaloids, while *G. applanatum* and *Flammulina velutipes* showed medium-dependent effects enhanced by wine yeast (Liew et al., 2015; Fidler et al., 2015). *Ganoderma* mycelium extracts outperformed fruiting bodies with lower MIC values against pathogens (Sharma et al., 2015). *G. lucidum*-enriched soap demonstrated antibacterial activity against *S. aureus* and antioxidant capacity (IC_50_ 1.53 mg/mL) (Hayati et al., 2020). *G. resinaceum* methanol extracts showed significant antioxidant and antimicrobial potential (Zengin et al., 2015), corroborated by other studies (Hoque et al., 2015; Kirar et al., 2015). *G. applanatum* methanolic extracts inhibited *S. typhi* (3.21 mm ZOI) and *P. mirabilis* (3.02 mm ZOI), containing phenolics (20.81 mg/100 g) and flavonoids (23.89 mg/100 g), with nutritional analysis revealing 222.08 Kcal/100 g and 42.72% carbohydrates (Dandapat et al., 2016).

Recent studies have demonstrated significant antimicrobial and antioxidant properties in various *Ganoderma* species. *G. lucidum* showed strongest inhibition against *Candida glabrata* (25 ± 1 mm) compared to *C. albicans* and *B. subtilis* (10 ± 1 mm), with its methanolic extract exhibiting exceptional DPPH radical scavenging activity (IC_50_ = 3.82 ± 0.04 μg/mL) attributed to phenolic compounds (Celık et al., 2014). Ethanol mycelial extracts of *Ganoderma* species, particularly *G. lucidum* BEOFB 433, displayed both antibacterial effects and antifungal activity against *Aspergillus glaucus* and *Trichoderma viride* (Ćilerdžić et al., 2016a). *Ganoderma pfeifferi* volatile oil, containing 73.6% 1-octen-3-ol, showed strong antimicrobial activity against *S. aureus* and *C. albicans* along with significant antioxidant capacity (Al-Fatimi et al., 2016), while *G. lucidum* fermentation broths demonstrated 39.67% antioxidant activity, with strain BEOFB 432 being particularly effective (Ćilerdžić et al., 2016b). Kenyan *G. lucidum* extracts exhibited activity against MRSA and common bacteria (up to 10.0 mm inhibition), highlighting its antimicrobial potential (Reid et al., 2016; Sande and Baraza, 2019). *Ganoderma tropicum* showed promise as a biocide and corrosion inhibitor against sulfate-reducing bacteria in industrial applications (Stanley et al., 2016). Comparative studies of eight mushroom species revealed that *G. applanatum*, *Laetiporus sulphureus*, *F. velutipes*, *Trametes versicolor*, *Hericium coralloides*, and *Agaricus campestris* had significant antimicrobial activity against *B. subtilis* ATCC 6633, while *G. lucidum* and *Pleurotus eryngii* showed no effects (Nicolcioiu et al., 2017). However, *G. lucidum* culture broth demonstrated antibacterial activity against *Staphylococcus epidermidis* and *P. aeruginosa*, suggesting potential for cosmetic and nutraceutical applications (Sarnthima et al., 2017).

GC-MS analysis of *G. lucidum* mycelia and fruiting bodies revealed that the mycelial aqueous extract possessed the highest anti-*Candida* activity (against *C. albicans* and *C. glabrata* biofilms) and ascorbic acid content, suggesting biofilm prevention potential. Chemometric analysis showed variability in volatile organic compounds between extracts (Bhardwaj et al., 2017). Antimicrobial testing of *G. lucidum* (GL) showed MICs of 200–400 µg/mL against *S. aureus*, *E. faecalis*, *L. monocytogenes*, *K. pneumoniae*, *P. aeruginosa*, *E. coli*, and *Candida* spp. While non-cytotoxic to NIH3T3 cells, GL showed genotoxicity (2.71-fold genetic damage at 5 mg/mL) (Ergun, 2017). *G. lucidum* ethanol extracts showed superior antibacterial activity (lower MICs against *S. aureus*, *M. luteus*, *B. terom*, and *B. subtilis*), while water extracts had higher DPPH scavenging (56.22% vs. 20.67%) (Wang et al., 2017). Philippine *G. applanatum* and *G. lucidum* ethanol extracts inhibited *S. aureus* (6.55 ± 0.23 mm to 7.43 ± 0.29 mm zones) with MIC_50_ values of 1,250–10,000 μg/mL (Gaylan et al., 2018). *G. lucidum* extract inhibited MDR *Mycobacterium tuberculosis* (complete inhibition at 25%–50% concentration) (Erawati et al., 2018). Bangladeshi *G. lucidum* exhibited antioxidant activity (IC_50_ 89.05 µg/mL), cytotoxicity (LC_50_ 142.49 µg/mL), and antibacterial effects against antibiotic-resistant strains (Islam et al., 2018). Antimicrobial peptides from *G. lucidum* fruiting bodies (GLF) and mycelium (GLM) showed activity against *E. coli* and *S. typhi* via ROS and protein leakage mechanisms (Mishra et al., 2018a). *G. lucidum*-based Kombucha beverage achieved 22.8 g/L acidity by day 2, with strong antioxidant/antibacterial activity (especially against *S. epidermidis* and *R. equi*), though the vacuum-dried form was less potent (Sknepnek et al., 2018).

Australian *G. lucidum* extracts demonstrated significant wound-healing properties, with ethanol/methanol-extracted triterpenes and water-extracted polysaccharides (50 mg/mL) showing antimicrobial activity against *S. aureus* (including MRSA), *B. cereus*, *S. pyogenes*, and *E. coli*. Alkali-extracted compounds were effective against *P. aeruginosa* (Montalbano, 2018). In food preservation, sausages with 0.5% *G. lucidum* powder maintained lower lipid oxidation and microbial levels while matching sensory acceptability of conventional preservatives (Ghobadi et al., 2018). *Ganoderma lipsiense* extract specifically inhibited *P. aeruginosa* (via phenolic compounds like caffeic acid) but not *E. coli* or *S. aureus* (Costa et al., 2019). Turkish *G. lucidum* exhibited high antioxidant potential (TAS/TOS/OSI assays) and antimicrobial activity against nine pathogens (Celal, 2019). Ethanol extracts (20 g/mL) showed the strongest activity against *S. aureus*, *P. aeruginosa*, and *Fusarium* sp (Tamilselvan and Rajesh, 2019). Serbian *Ganoderma* species revealed species-specific efficacy: *G. resinaceum* chloroform extract against *P. aeruginosa*, *G. pfeifferi* water extract against *E. coli*/*S. aureus*, and *G. lucidum* showing antiviral potential (Rašeta et al., 2023). South Jakarta *G. lucidum* ethanol extract only affected *S. aureus*, with no dose-dependent improvement (Noverita and Ritchie, 2020). GC-MS analysis of Nigerian *G. lucidum* identified 48 bioactive compounds (including BHA), with methanol extracts showing the strongest antibacterial effects (except against resistant *P. aeruginosa*) (Anyakorah et al., 2020). Kenyan studies confirmed *G. lucidum* and *Termitomyces letestui* activity against MRSA and *S. pyogenes* (Anyimba, 2020). Methanol extracts from Yeast Wine Media completely inhibited fungal growth (500–1,000 ppm) and showed superior activity against *Xanthomonas oryzae*/*Ralstonia solanacearum*, with higher antioxidant capacity (Suansia and John, 2021). Finally, *G. lucidum* methanol extracts exhibited potent antibacterial effects against MDR *E. coli* and *P. aeruginosa* (19.3 ± 0.4 mm zones, MBC 266 ± 23.6) (Radhika, 2021).

Comparative analysis of *G. lucidum* mycelium and spores against *P. intermedia* from periodontitis patients revealed mean MIC values of 5.64 mcg/mL (mycelium) and 3.62 mcg/mL (spores), demonstrating comparable antimicrobial efficacy for adjunct periodontal therapy (Nayak et al., 2021). Mexican *G. curtisii* strains exhibited notable biological activities, including tumor cell line inhibition (GI_50_ ≤50 µg/mL), anti-*S. aureus* effects, and antioxidant properties, with strain GH-16–023 showing particularly low toxicity (Serrano-Márquez et al., 2021). Kenyan *G. lucidum* extracts contained terpenoids, phenolics, and glycosides, displaying significant activity against MRSA and *Streptococcus pyogenes*, with the isolated compound Ergosta-5,7,22-triene-3β,14α-diol showing potent antibacterial effects (Baraza et al., 2021). *G. lucidum* spore powder aqueous extracts demonstrated remarkable antibacterial activity with MIC values of 125 µg/mL (*S. aureus* and *E. coli*), <2 µg/mL (*E. faecalis*), and 62.5 µg/mL (*K. pneumoniae*) (Nayak et al., 2010a). Comparative studies of *G. boninense* extracts revealed that chloroform-extracted mycelium (GBMA) exhibited the strongest antibacterial activity, particularly through chloroform-methanol-water extraction, suggesting novel antimicrobial metabolites (Abdullah et al., 2020). Further analysis of *G. boninense* fruiting bodies showed that ethyl acetate extracts had broad-spectrum inhibition (especially against *P. mirabilis*), while methanol extracts showed the lowest MIC (0.625 mg/mL) against Coagulase-Negative Staphylococci, with LC-MS identifying alkaloids, fatty acids, and glycosides as potential bioactive compounds (Chan and Chong, 2020).

Medicinal polypores including *G. adspersum*, *G. applanatum*, and *G. australe* yielded bioactive ergostane compounds (ergosta-7,22-dien-3-one and ergosta-7,22-diene-3β-ol) through methanol/ethyl acetate extractions, showing significant inhibition against *S. pyogenes* but not Gram-negative bacteria, suggesting potential for novel myco-medicines (Mayaka, 2020). In biofilm-related studies, *G. lucidum* demonstrated notable anti-biofilm activity against multidrug-resistant (MDR) *Enterococcus* strains, offering alternatives for challenging infections (Karaca et al., 2020). Phytochemical analysis revealed that wild *Ganoderma* species contained saponins and flavonoids, with *G. lucidum* showing the highest cyanide content. Ethanolic extracts inhibited *Salmonella* spp., *E. coli*, *S. aureus*, and *Streptococcus* spp., with *G. applanatum* particularly effective against *E. coli* (19.50 mg/mL) and all species showing similar MBC (~250 mg/mL) (Wood et al., 2021). Optimized cultivation of Philippine *G. lucidum* on sawdust/PDA yielded ethanol extracts (100–200 mg/mL) that outperformed standard antibiotics in antibacterial tests, with fruiting bodies showing superior antioxidant activity to mycelia (Subedi et al., 2021). Nine *Ganoderma* species extracts, including *G. tuberculosum* and *G. tornatum*, inhibited *Clavibacter michiganensis* (31.5–1,000 μg/mL), suggesting applications for tomato canker management (Espinosa-García et al., 2021).

Comparative studies of medicinal mushrooms revealed that *Taiwanofungus camphoratus* methanolic extracts showed strong antimicrobial activity, while *G. lucidum* extracts displayed no significant effects, with concerns about *Penicillium expansum* developing tolerance (Kim et al., 2022). *G. boninense* demonstrated exceptional anti-MRSA activity (41.08 mm zone, MIC 0.078 mg/mL) through membrane disruption, with LC-MS identifying eight bioactive compounds (Chan and Chong, 2022). Iraqi studies showed that *G. lucidum* methanol extract (200 mg/mL) was most effective against UTI pathogens (*K. pneumoniae*, *S. aureus*, and *P. mirabilis*), containing flavonoids, alkaloids, phenols, and terpenoids (Shawkat and Aedan, 2022). Metabolite profiling of six *Ganoderma* species identified *G. pfeifferi* as the richest in phenolic acids (114.07 mg/100 g DW) and *G. lucidum* as the richest in indole compounds, with all showing antioxidant and enzyme inhibitory potential (Sułkowska-Ziaja et al., 2022). *G. lucidum* methanol extract demonstrated dual anti-MRSA activity *in vitro* and *in vivo*, reducing lung inflammation and LDH levels in infected rats (Soliman et al., 2022). Moroccan studies revealed the potent antimicrobial activity of *G. lucidum* extract (especially against *Epidermophyton floccosum*) and high phenolic/flavonoid content (Erbiai et al., 2023). Further studies confirmed antimicrobial (MIC 50 μg/mL against *S. aureus/E. coli*) and antioxidant (85.9%–90.12% radical scavenging at 400 μg/mL) properties of *G. lucidum* (Tehranian et al., 2023). Turkish specimens showed 90.81% DPPH scavenging and notable anti-*E. faecalis* activity (17.67 ± 0.47 mm zone), with GC-MS identifying key fatty acids (Canpolat and Canpolat, 2023). *Ganoderma mbrekobenum* methanol extracts showed strong anti-*Bacillus* (15- to 18-mm zones) and anti-*Fusarium* activity, with 46 bioactive compounds identified (El-Dein et al., 2023). Antifungal studies demonstrated that *G. lucidum* pure extract achieved 100% inhibition of *Colletotrichum gloeosporioides* and 94.4% against *Alternaria solani* (Saludares et al., 2023). Comparative analysis showed that *G. lucidum* surpassed *G. neo-japonicum* in protein content (24.3 vs. 15.6 mg/g), phenolics (14.3 vs. 9.8 mg GAE/g), and antioxidant capacity (FRAP 403.9 μmol Fe^2+^/g) (Ayimbila et al., 2023). The extraction method significantly influenced bioactivity—Soxhlet ethanol extracts showed strongest anticancer effects (MCF-7 IC_50_ 4.797 μg/mL) while UAE water extracts had the best anti-*S. aureus* activity (20–23 mm) (Azahar et al., 2023).

#### 5.1.2 Triterpenoids

Infectious diseases caused by bacteria, fungi, viruses, and parasites remain a leading cause of global morbidity and mortality, particularly in low- and middle-income countries. The rise of AMR, emerging viral pathogens, and neglected tropical diseases underscores the urgent need for new therapeutic agents. *Ganoderma* species, especially through their triterpenoid-rich extracts, represent a promising yet underutilized resource in addressing these critical health challenges. Triterpenoids, particularly lanostane-type compounds, are among the most bioactive secondary metabolites in *Ganoderma* spp., exhibiting broad-spectrum antimicrobial and antiviral activity (**Table 1**). Their multifaceted mechanisms include membrane disruption, enzyme inhibition, and immunomodulation.

**Table 1:** Antimicrobial properties of triterpenoids in *Ganoderma* species.

Species	Key compounds/extracts	Key findings	Activity indicator	Disease relevance/Target pathogens	References
*Ganoderma lucidum*	Ganoderic acids GA-T and GA-Me	Antibacterial and antifungal activity	MIC: 150 µg/mL (bacteria), 100 µg/mL (fungi)	Potential use in treating dermatomycoses, respiratory infections, and Gram-positive sepsis	Shveta et al., 2013
	Triterpenoid extract from GLSP	Inhibits *S. aureus* and *E. coli*	61.09% DPPH inhibition	Relevance to skin and urinary tract infections (UTIs)	Shen et al., 2020
	Ethanolic extract (lanostanoid ester)	Active against *S. aureus* and *B. subtilis*	MIC 68.5 µM (*S. aureus*), 123.8 µM (*B. subtilis*)	Relevance to hospital-acquired infections	Liu et al., 2014
*G. applanatum*	Lanostanoids, sterols	Broad antibacterial spectrum	MIC: 0.003–2.0 mg/mL; MBC: 0.06–4.0 mg/mL	Targets respiratory tract bacteria; potential for topical wound infections	Smania et al., 1999
	Lanostane triterpenoids	Notable antimicrobial effects	<60 μg/mL	Relevance to cutaneous fungal infections	Shi et al., 2022
*G. casuarinicola*	Norlanostanes, ganocasuarinone A	Active against *S. aureus* and *M. tuberculosis*	5 mg/mL (*S. aureus*), 25–50 µg/mL (*M. tuberculosis*)	Relevance to tuberculosis and Gram-positive infections	Isaka et al., 2020

Early studies on *G. applanatum* identified three sterols and a novel lanostanoid with potent antibacterial activity, showing Gram-positive specificity (MIC: 0.003–2.0 mg/mL; MBC: 0.06–4.0 mg/mL) (Smania et al., 1999). Nigerian *G. colossum* yielded new colossolactones including 23-hydroxycolossolactone E with antimicrobial potential (Ofodile et al., 2005). Modified applanoxidic acids from *Ganoderma* spp. maintained activity against *E. coli*, *S. aureus*, *C. albicans*, and *T. mentagrophytes* (MIC: 1.0 to >2.0 mg/mL) (Smania et al., 2006). Western Ghats *Ganoderma* sesquiterpenoids surpassed standard antibiotics against bacteria and *C. albicans*, while triterpenes showed weaker effects (Bhosle et al., 2010). Colossolactones E and 23-hydroxycolossolactone E demonstrated activity against *B. subtilis* and *P. syringae* (Ofodile et al., 2011), with *G. lucidum* and *G. mazandaran* showing the lowest MICs (128 µl/mL) against *B. subtilis* and *P. mirabilis* (Ofodile et al., 2012). Haryana *G. lucidum* yielded ganoderic acids (GA-T and GA-Me) with MICs of 150 µg/mL (bacteria) and 100 µg/mL (fungi) (Shveta et al., 2013). *Ganoderma* sp. BCC 16,642 produced ganoderic acids/lanostanoids active against *S. aureus* and *B. subtilis* (Liu et al., 2014). Ethyl acetate extracts of *G. lucidum* contained novel antimicrobial triterpenoids (Liu et al., 2014), while its GA showed cytotoxicity and antibacterial effects (Upadhyay et al., 2014). Two triterpenoids (GLTA and GLTB) exhibited anti-EV71 activity by blocking viral adsorption and RNA replication (Zhang et al., 2014). *Ganoderma* triterpenoids inhibited *S. aureus* biofilms and *E. coli* (Basnet et al., 2017). Recent studies revealed that *G. lucidum* spore powder triterpenoids had 61.09% DPPH inhibition (600 µg/mL) and anti-*S. aureus/E. coli* activity (Shen et al., 2020). *Ganoderma casuarinicola* norlanostanes showed anti-*S. aureus* (5 mg/mL) and anti-TB (25–50 µg/mL) effects (Isaka et al., 2020). *G. applanatum* yielded three new antimicrobial lanostane triterpenoids (Shi et al., 2022). Given their demonstrated efficacy against MDR bacteria (e.g., MRSA), biofilm-producing strains, and even viruses, *Ganoderma*-derived triterpenoids offer a compelling lead for drug discovery targeting difficult-to-treat infections. Their ability to address current gaps in antifungal and antiviral therapeutics, coupled with favorable safety profiles in traditional use, reinforces their potential for clinical translation. **Table 2** provides an overview of the antibacterial properties exhibited by various extracts of Ganoderma species.

**Table 2:** Overview of antibacterial properties in *Ganoderma* extracts.

Species	Extract type	Key findings	Potential applications/Disease relevance	References
*Ganoderma applanatum*	Methanol/Methanolic/Ethanolic	Strong activity against Gram-positive bacteria and some fungi; phenolic-rich	Potential treatment for skin infections, respiratory infections, and Gram-positive sepsis in humans and animals	Pushpa et al., 2013; Moradali et al., 2008; Dandapat et al., 2016; Rijia et al., 2024; Gaylan et al., 2018
	Extracts	Highest antibacterial and antifungal activity	Potential for broad-spectrum antimicrobial therapies	Lone et al., 2024
*G. boninense*	Methanol/Ethyl acetate/Chloroform	Broad-spectrum activity, including MRSA; membrane disruption	Wound infections, multidrug-resistant bacterial infections	Ismail et al., 2014; Chan and Chong, 2020, 2022; Abdullah et al., 2020
*G. carnosum*	Dichloromethane extracts	Antibacterial and antifungal; antioxidant properties	Topical antimicrobials, antifungal creams, plant protection	Srivastava and Sharma, 2011; Sułkowska-Ziaja et al., 2022
*G. colossus*	Dichloromethane, Methanolic, Water	Effective against *E. coli* and *S. aureus*	Gastrointestinal and skin infections	Al-Fatimi et al., 2005
*G. curtisii*	Extracts	Antiproliferative, antioxidant, and antibacterial effects	Immunocompromised patient care, supportive cancer therapy	Serrano-Márquez et al., 2021
*G. lucidum*	Multiple solvents	Broad antimicrobial activity; quorum sensing inhibition	Anti-biofilm agent in chronic respiratory or wound infections	Fakoya et al., 2013; Gowrie et al., 2014; Shang et al., 2013; Zhu et al., 2011; others
*G. tsugae*	Chitosan extracts	Strong antibacterial, surpassing doxycycline	Acne treatment, resistant skin infections	Chen et al., 2007
*G. resinaceum*	Dichloromethane, Methanolic, Water	Active against several bacterial pathogens	Alternative to conventional antibiotics	Al-Fatimi et al., 2005
*G. tuberculosum*, *G. tornatum*, *G. weberianum*	Chloroform-methanol extracts	Antibacterial against *Clavibacter michiganensis*	Crop disease biocontrol (e.g., tomato canker)	Espinosa-García et al., 2021
*Ganoderma* spp.	Various solvents	Antibacterial and antifungal against human/plant pathogens	Agricultural biopesticide or general therapeutic candidate	Yamac and Bilgili, 2006

#### 5.1.3 Polysaccharides

Polysaccharides from *Ganoderma* species, particularly *G. lucidum*, offer compelling bioactivity that aligns with global efforts to combat infectious diseases. As AMR and gastrointestinal infections continue to rise globally, especially in immunocompromised populations and developing regions, the need for non-antibiotic, immune-enhancing alternatives becomes critical. *Ganoderma*-derived polysaccharides, rich in β-glucans and heteropolysaccharides, are emerging as promising immunomodulatory and antimicrobial agents that could complement or replace conventional antimicrobials (**Table 3**).

**Table 3:** Polysaccharides in *Ganoderma* species and their antimicrobial properties.

*Ganoderma* species	Polysaccharide composition	Pathogens targeted	Disease relevance/Target infection	References
*G. lucidum*	D-glucose-based polysaccharides	Plant and foodborne microbes	Foodborne infections, gastrointestinal illness	Bai et al., 2008
	Polysaccharides	Gram-positive bacteria	Skin infections, respiratory pathogens	Skalicka-Wozniak et al., 2012
	Polysaccharides	Bacterial pathogens	General bacterial infections in humans	Batra and Khajuria, 2012
	Exopolysaccharides (EPS)	*Bacillus cereus*	Food poisoning, diarrheal syndromes	Mahendran et al., 2013
	Polysaccharides	Opportunistic bacteria	Hospital-acquired infections (e.g., wound and lung)	Kaur et al., 2015
	(1,3)-β-D-glucan, GS	Foodborne and clinical strains	Enteric infections, sepsis-related strains	Wan-Mohtar et al., 2016
	Chitosan	Gram-positive cocci	Skin and bloodstream infections (e.g., *S. aureus*)	Savin et al., 2020
	Low-MW polysaccharides (3.5–4.5 kDa)	Agricultural pathogens	Zoonotic bacterial risks through crops	Robles-Hernández et al., 2021
	Polysaccharides	*E. coli* strain	Gastrointestinal infections and UTIs	Zhai et al., 2021
*G. multicornum*, *G. multiplicatum*, *G. perzonatum*, and *G. stipitatum*	Polysaccharides	Enteric bacteria	Diarrheal diseases in livestock and humans	Sharifi et al., 2012
Various *Ganoderma* spp.	Polysaccharides	Mixed bacterial species	Broad-spectrum infections (foodborne, respiratory)	Al-Fatimi et al., 2005; Isaka et al., 2016

Hot water extracts of *G. lucidum* fruiting bodies, primarily composed of D-glucose, have demonstrated activity against plant pathogens (*Erwinia carotovora* and *Penicillium digitatum*) and foodborne microbes (*B. cereus*, *E. coli*, and *Aspergillus niger*) (Bai et al., 2008). *G. lucidum* polysaccharides also strongly inhibit *M. luteus* (MIC 0.62–1.25 mg/mL) (Skalicka-Wozniak et al., 2012), and fractions isolated from *G. multicornum* and related species show activity against *E. coli* and *P. mirabilis* (Sharifi et al., 2012). Additional studies revealed inhibition zones up to 19 mm against *Staphylococcus* sp. (Batra and Khajuria, 2012) and potent activity (18- to 23-mm inhibition zones) from exopolysaccharides (EPS) cultivated on basal and malt media (Mahendran et al., 2013). Strain-specific studies showed that *G. lucidum* GL-2 and GL-3 produce polysaccharides that inhibit *Staphylococcus* and *Enterobacter* spp (Kaur et al., 2015). Mechanistically, these polysaccharides exert their antimicrobial action by disrupting microbial cell walls and modulating oxidative stress. Their synergy with phenolic compounds enhances antimicrobial efficacy, suggesting a multi-targeted mode of action (Al-Fatimi et al., 2005; Isaka et al., 2016).

*G. lucidum* strain BCCM 31549 produces both (1,3)-β-D-glucan (G) and its sulfated derivative (GS), with GS exhibiting not only superior antimicrobial activity but also selective cytotoxicity against *U937* cancer cells (Wan-Mohtar et al., 2016), pointing to potential dual anti-infective and anticancer utility. Enzymatically extracted chitosan from *G. lucidum* shows superior antibacterial effects against Gram-positive bacteria and improved antioxidant activity compared to chemically extracted counterparts (Savin et al., 2020). Small-molecular-weight polysaccharides (3,500–4,500 Da) isolated from culture fluids have recently demonstrated strong antibacterial effects against plant pathogens (Robles-Hernández et al., 2021), offering a sustainable source for agricultural biocontrol. In a more clinically relevant context, *G. lucidum* polysaccharides at concentrations of 5–100 μg/mL not only inhibited *E. coli* proliferation but also modulated immune response pathways in intestinal porcine epithelial cells (IPEC-1), suggesting potential for treating or preventing bacterial gut infections (Zhai et al., 2021).

Collectively, these findings suggest that *Ganoderma* polysaccharides can address important global health challenges such as antibiotic-resistant bacterial infections, especially gastrointestinal and foodborne diseases. Their natural origin, immunostimulatory properties, and low toxicity support their further development as functional antimicrobial agents or as adjuncts to conventional therapies.

#### 5.1.4 Other compounds

In addition to triterpenoids and polysaccharides, *Ganoderma* species produce a chemically diverse repertoire of secondary metabolites—including essential oils, steroids, phenolics, alkaloids, and proteins—that contribute to their antimicrobial properties (**Table 4**). These compounds are increasingly viewed as promising leads in the search for novel anti-infective agents, particularly against MDR pathogens. Given the growing global burden of AMR, notably *S. aureus*, *M. tuberculosis*, and nosocomial Gram-negative infections, such natural compounds represent a valuable reservoir for alternative therapies and adjunct treatments. Essential oils derived from *G. japonicum* mycelia, rich in nerolidol and linalool, exhibited potent activity against MRSA, with a minimum bactericidal concentration (MBC) of 1.03 mg/mL (Liu et al., 2009). *G. pfeifferi* produced ganomycins A and B, which demonstrated pronounced anti-Gram-positive activity (MIC 2.5–25 µg/mL) (Mothana et al., 2000), suggesting potential as topical agents or adjuvants for skin and wound infections. Novel metabolites from *G. australe*, including australic acid, showed broad-spectrum antimicrobial effects (Smania et al., 2007), while solvent extracts of *G. lucidum* yielded terpenoids, alkaloids, and steroids with wide-ranging antimicrobial activity (Subbraj et al., 2008). Proteinaceous extracts from *G. resinaceum* also demonstrated notable activity against hospital-associated pathogens, including *E. coli*, *S. aureus*, and *K. pneumoniae* (Hearst et al., 2010), while *G. lucidum* extracts produced inhibition zones up to 16 mm against MDR clinical isolates (Sekaran et al., 2011). Steroidal compounds from several *Ganoderma* species were shown to inhibit *M. tuberculosis* (MIC 0.781–50 µg/mL) and Gram-positive cocci (Vazirian et al., 2014), underscoring their relevance for neglected and resurgent infectious diseases such as tuberculosis. Innovative processing and analytical techniques have recently advanced the identification of bioactives from *Ganoderma*. Gamma irradiation enhanced the antimicrobial potency of *G. resinaceum* (Abd El-Zaher, 2010), while LC-MS analysis detected bioactive compounds such as hesperetin and ganocin B in *G. lucidum* (Abdullah et al., 2021). Optimized extraction protocols yielded phenolic-rich fractions (16.01 mg/g total phenolics) from *G. lucidum*, which showed potent activity against *S. aureus* (10.6-mm inhibition zone) (Masjedi et al., 2022). These effects are partly attributed to ROS-mediated bacterial protein leakage, as evidenced by phenolic fractions of *G. lucidum* (Mishra et al., 2018b). Moreover, uncooked *Ganoderma* biomass has shown dual antimicrobial and anticancer activity, offering potential for functional food or nutraceutical applications (Alghonaim et al., 2023). Altogether, these studies reveal that non-triterpenoid *Ganoderma* metabolites—especially essential oils, phenolics, and proteins—may offer novel solutions to combat AMR and opportunistic infections. However, their clinical translation remains limited due to a lack of *in vivo* validation, pharmacokinetic profiling, and toxicity assessments. Future work should prioritize preclinical testing of these compounds in infection models, particularly for high-burden diseases such as tuberculosis, hospital-acquired infections, and drug-resistant enteric pathogens.

**Table 4:** Antimicrobial properties of various other compounds isolated from *Ganoderma* species.

*Ganoderm*a sp.	Main bioactive components	Pathogens targeted	Disease relevance/Target infection	References
*Ganoderma atrum*	Sterols	Oxidative protection in Caco-2 cells	Intestinal epithelial protection, gut inflammation	Guo et al., 2022
*G. australe*	Australic acid and methyl australate	Gram-positive and Gram-negative bacteria, fungi	Broad-spectrum antimicrobial for skin and internal infections	Smania et al., 2007
*G. boninense*	Ergosterol and ganoboninketals	*S. aureus* strains	Skin infections, pneumonia, endocarditis	Abdullah et al., 2021
*G. japonicum*	Nerolidol, linalool, decadienal, and benzyl alcohol	18 microorganisms, especially MRSA	Multidrug-resistant infections (e.g., MRSA in hospitals)	Liu et al., 2009
*G. lucidum*	Steroids, terpenoids, and alkaloids	Gram-positive bacteria	Respiratory and skin infections	Subbraj et al., 2008
	Phenolic compounds	Pathogenic bacteria	General bacterial infections	Mishra et al., 2018b
	Tannins, phenolics, flavonoids, and saponins	*P. aeruginosa*, *E. coli*, *S. aureus*, and *K. pneumoniae*	Wound infections, UTIs, and nosocomial pathogens	Sekaran et al., 2011
	Uncooked biomass	Antimicrobial and anticancer (MCF-7 cells)	Breast cancer and general microbial infection	Alghonaim et al., 2023
*G. resinaceum*	Peptides	*E. coli*, MRSA, and *Salmonella*	Gastrointestinal and systemic infections	Hearst et al., 2010
	Lipids	*Fusarium oxysporum* and *Candida albicans*	Mycotic infections in humans and animals	Abd El-Zaher, 2010
*Ganoderma* spp.	Ganomycins A and B	*S. aureus* and *Micrococcus flavus*	Gram-positive infections in skin and soft tissue	Mothana et al., 2000
	Steroidal compounds	*Mycobacterium tuberculosis*, *S. aureus*, and *B. subtilis*	Tuberculosis and staph-related infections	Vazirian et al., 2014
	Multiple compounds	*P. aeruginosa*, *S. typhimurium*, and *K. pneumoniae*	GI, respiratory, and opportunistic infections	

#### 5.1.5 Nanoparticles

The global rise of AMR and chronic biofilm-associated infections underscores the urgent need for novel, multi-targeted therapeutics that are both effective and sustainable. Nanotechnology has emerged as a powerful tool in this arena, and *Ganoderma*-derived nanoparticles—particularly silver nanoparticles (Ag-NPs)—represent a promising frontier in fungal biomedicine. Infections caused by MDR pathogens such as *S. aureus*, *E. coli*, and *P. aeruginosa* remain major contributors to mortality in hospitals worldwide, with the WHO designating these as “priority pathogens.” Numerous studies have demonstrated that Ag-NPs synthesized from *G. lucidum*, *G. resinaceum*, and *G. sessile* exhibit broad-spectrum antibacterial activity, often surpassing the efficacy of conventional antibiotics or potentiating their effects through synergistic mechanisms (Kannan et al., 2014; Ali Syed et al., 2023; **Table 5**).

**Table 5:** Overview of antimicrobial activity and applications of nanoparticles derived from *Ganoderma* species.

*Ganoderm*a species	Nanoparticle type	Antimicrobial activity	Additional applications	References
*G. lucidum*	Silver (Ag-NPs)	Active vs. *S. aureus*, *E. coli*, and *P. aeruginosa*; enhances antibiotics	Therapeutic, anticancer (IC_50_ 9.2 µg/mL), wound dressings, and public health	Kannan et al., 2014; Al-Ansari et al., 2020; Paul et al., 2015; Constantin et al., 2023
	Polysaccharide NPs	Improved antimicrobial and antioxidant activity	Drug delivery	Qin et al., 2018
	Modified sodium montmorillonite	Corrosion resistance and hydrophobicity	Nanocomposites	Sheydaei et al., 2023
*G. applanatum*	Silver (Ag-NPs)	Active vs. *E. coli* and *S. aureus*	Biomedical applications	Mohanta et al., 2016; Jogaiah et al., 2019
*G. sessiliforme*	Silver (Ag-NPs)	Effective vs. foodborne pathogens	Antioxidant and cytotoxic effects	Mohanta et al., 2018
	Copper oxide (CuONPs)	Active vs. *S. aureus*, *E. coli*, and *P. aeruginosa*	Treatment of superficial infections	Flores-Rábago et al., 2023
*G. sessile*	Metallic NPs	Active vs. *Campylobacter jejuni*	Foodborne illness control	Rivera-Mendoza et al., 2024
*G. resinaceum*	Silver (Ag-NPs)	Active vs. multidrug-resistant pathogens	—	Ali Syed et al., 2023
*G. boninense*	Phenolic compounds	Strong fungitoxicity	—	Chong et al., 2011
*Ganoderma* spp.	Titanium dioxide (TiO_2_) NPs	Effective vs. biofilm-forming pathogens	Clinical antibacterial agents	Marzhoseyni et al., 2023
	Silver nanocomplex	Broad-spectrum bactericidal	Eco-friendly antimicrobial agent	Shokouhi et al., 2023

In resource-limited settings where access to antibiotics is restricted, these green-synthesized nanoparticles offer a cost-effective and scalable antimicrobial alternative. Their ability to disrupt bacterial membranes, generate ROS, and inhibit efflux pumps suggests utility in treating persistent infections such as those found in tuberculosis, diabetic wounds, and catheter-associated UTIs (Al-Ansari et al., 2020; Bhardwaj et al., 2016). Moreover, the low toxicity of *Ganoderma*-synthesized copper oxide nanoparticles (CuONPs) supports their potential use in topical formulations for superficial infections, particularly in low-income regions (Flores-Rábago et al., 2023).

Importantly, *Ganoderma*-derived nanoparticles also show activity against biofilm-forming pathogens, a major clinical challenge in implant-related infections and chronic wounds. Biofilms protect microbes from host immunity and antibiotics, contributing to prolonged hospital stays and increased mortality. Titanium dioxide nanoparticles combined with *Ganoderma* extracts have shown antibiofilm efficacy, which could be leveraged in medical device coatings and sterile wound dressings (Marzhoseyni et al., 2023; Paul et al., 2015). The anticancer and antioxidant properties of these nanoparticles add another layer of relevance. Ag-NPs synthesized from *G. lucidum* and *G. sessiliforme* have demonstrated cytotoxicity against breast and lung cancer cell lines, potentially addressing cancer-related infections and immune suppression (Mohanta et al., 2018; Bhardwaj et al., 2016). In cancer patients with neutropenia or post-chemotherapy immune suppression, fungal or bacterial co-infections are common. Thus, dual-action nanoparticles offer a novel approach to oncological support therapy.

In food safety and agriculture, *Ganoderma*-based nanoparticles have been tested against *Campylobacter jejuni*, a major cause of gastroenteritis and post-infectious sequelae in developing nations (Rivera-Mendoza et al., 2024). This points to a broader public health application, particularly in addressing foodborne diseases and improving sanitation in regions with limited access to refrigeration or clean water. Although current studies are predominantly *in vitro*, the eco-friendly synthesis, scalability, and multipotent biological activities of *Ganoderma*-derived nanoparticles position them as strong candidates for next-generation antimicrobials. Future work must address *in vivo* efficacy, targeted delivery mechanisms, pharmacokinetics, and regulatory considerations to facilitate clinical translation. Hence, *Ganoderma*-based nanomaterials not only show promise against MDR pathogens and biofilms but also align with global health priorities such as reducing AMR, treating co-infections in cancer or HIV patients, and improving access to antimicrobial materials in underserved regions. These properties highlight their unmet therapeutic potential in both developed and developing healthcare systems.

### 5.2 Fungal infections

Fungal infections pose a growing threat to global health, particularly among immunocompromised individuals, transplant recipients, and patients undergoing chemotherapy. According to the Global Action Fund for Fungal Infections, over 1.5 million deaths annually are attributed to invasive fungal diseases, and current treatments are limited by toxicity, poor bioavailability, and rising resistance—especially in *Candida* and *Aspergillus* species. The pipeline for new antifungal drugs remains dangerously sparse, underlining the urgent need for novel, safer, and more effective agents. Against this backdrop, *Ganoderma* species, particularly *G. lucidum*, offer promising antifungal potential with mechanisms distinct from conventional agents (**Table 6**). *G. lucidum* has demonstrated broad-spectrum activity against pathogenic fungi, including *C. albicans*, *Aspergillus flavus*, and *Fusarium oxysporum*, with some studies reporting MIC values below 1 µg/mL (Bitew and Abate, 1994). These findings are not merely academic; they suggest that *Ganoderma*-derived compounds could fill critical therapeutic gaps in treating drug-resistant candidiasis and aspergillosis, which are common and often fatal in ICU patients and those with hematological malignancies.

**Table 6:** Antifungal compounds and activities of various *Ganoderma* species against pathogenic fungi.

Species	Antifungal compound	Target pathogen	Disease relevance/Target infection	References
*G. lucidum*	Culture filtrate	*Candida albicans*	Candidiasis (oral, vaginal, and systemic)	Bitew and Abate, 1994
	Ganodermin	Plant and postharvest fungi	Agricultural applications (not animal/human-specific)	Wang and Ng, 2006
	Toothpaste formulation	Oral *Candida*	Oral candidiasis and dental hygiene	Dzubak et al., 2006; Nayak et al., 2010b
	Acetone extract	Filamentous fungi	Respiratory or skin mycoses	Singh et al., 2014
	Methanolic extracts	Soil and plant-associated fungi	Opportunistic infections in immunocompromised hosts	Baig et al., 2015
	Ethanol and aqueous extracts	Opportunistic and phytopathogenic fungi	Human fungal infections (skin, respiratory); some plant relevance	Parkash and Sharma, 2016; Radhika and Rajan, 2021
	Glucan sulfate (GS)	*Aspergillus* spp.	Aspergillosis (pulmonary or systemic)	Wan-Mohtar et al., 2017
	PMMA modification	*Candida albicans*	Denture-related candidiasis	Enaba and El Gendi, 2022
	Triterpenoids	Dermatophytes and molds	Skin infections like ringworm and athlete’s foot	Wasser, 2011
	Secondary metabolites	Docked with *S. aureus* protein targets	Suggests dual antibacterial/antifungal action, relevant for mixed infections	Nguyen et al., 2024
	Ethanolic extracts	*Aspergillus flavus*	Food spoilage fungi and risk of aflatoxicosis in animals	Vahdani et al., 2022
*G. boninense*	Methanolic extracts	*Candida albicans*	Vulvovaginal and systemic candidiasis	Daruliza et al., 2012
*G. annulare*	Applanoxidic acids A, C, and F	Dermatophytes	Human skin infections (tinea and athlete’s foot)	Smania et al., 2003
*G. mbrekobenum*	Mycelial plugs	Feed-contaminating fungi	Prevention of mycotoxicosis in livestock	El-Fallal et al., 2021
*Ganoderma* sp.	Crude exopolysaccharides	Mixed fungal species	General antifungal for clinical and food safety uses	Demir and Yamaç, 2008
	Various extracts	Multiple human and plant pathogens	Broad antifungal; relevant for dermatological and respiratory infections	Migahed et al., 2018; Naveenkumar et al., 2018
	Not specified	*Aspergillus niger*	Opportunistic pathogen in immunocompromised individuals	Chandrawanshi and Shukla, 2019

Among the most notable bioactives is ganodermin, a protein isolated from *G. lucidum* that inhibits multiple phytopathogens (Wang and Ng, 2006), with potential for further development into topical antifungal formulations. In clinical contexts, *G. lucidum* has been incorporated into products like antifungal toothpaste and biomaterials such as polymethylmethacrylate (PMMA), where it enhanced mechanical performance while inhibiting *C. albicans* biofilm formation, a common cause of denture stomatitis (Nayak et al., 2010b; Enaba and El Gendi, 2022).

The unique mode of action of *Ganoderma*-derived triterpenoids—targeting ergosterol to disrupt fungal membranes—may offer an alternative to existing ergosterol-targeting drugs like amphotericin B but with lower toxicity (Wasser, 2011). In addition, these compounds have demonstrated the ability to interfere with biofilm formation and fungal cell wall synthesis, both of which are key contributors to antifungal resistance and treatment failure (Chan et al., 2013).

Importantly, *Ganoderma* extracts have shown efficacy against dermatophytes such as *Microsporum canis* and *Trichophyton mentagrophytes*, which are prevalent in tropical climates and often undertreated due to limited healthcare access (Smania et al., 2003). In veterinary and agricultural sectors, *Ganoderma* is also emerging as a natural antifungal for contaminated feed and crops, suggesting a One Health approach to fungal control (El-Fallal et al., 2021). From a pharmaceutical development perspective, molecular docking studies have revealed strong binding affinities of *Ganoderma* metabolites to key fungal protein targets, offering a rational basis for structure-based drug design (Nguyen et al., 2024). This computational insight strengthens the argument for clinical translation and underscores the need for further *in vivo* validation and toxicity profiling. Hence, the antifungal properties of *Ganoderma* are not just promising *in vitro* but potentially transformative in clinical settings where fungal infections are increasing and treatment options remain inadequate. By targeting resistant strains, disrupting biofilms, and offering low-toxicity alternatives, *Ganoderma*-derived compounds could represent the next generation of antifungal therapeutics—especially in settings where conventional options fall short.

### 5.3 Viral infections

Viral infections remain a major global health challenge, with diseases such as HIV/AIDS, hepatitis B (HBV), herpes simplex (HSV), and influenza collectively causing significant morbidity and mortality. According to UNAIDS, approximately 39 million people were living with HIV globally in 2023, while WHO reports over 250 million people chronically infected with HBV. These figures underscore the urgent need for novel antiviral agents, especially in light of emerging drug resistance and the limited efficacy or accessibility of current therapeutics in many regions. *Ganoderma* species, particularly *G. lucidum*, have garnered interest for their potential to address these unmet needs through their diverse arsenal of bioactive compounds (**Table 7**). Isolated triterpenoids, such as ganoderic acid-β, lucidumol B, and ganodermanontriol, have demonstrated significant anti-HIV-1 protease activity, with IC_50_ values ranging from 20 to 90 μM (Min et al., 1998; El-Mekkawy et al., 1998). Importantly, molecular docking studies suggest that ganoderic acid-B exhibits a binding affinity surpassing that of the standard drug nelfinavir, supporting its potential as a lead compound for drug development (Kang et al., 2015). In addition, enzymatic crude extracts rich in laccase from *G. lucidum* have shown remarkable *in vitro* inhibition of HIV-1 replication (Zhang et al., 2011; Flórez-Sampedro et al., 2016), providing an alternative strategy targeting reverse transcription pathways.

**Table 7:** Antiviral activity of compounds derived from *Ganoderma* species against various viral infections.

*Ganoderma* species	Active compound(s)	Target virus	Mechanism/Effect	Disease relevance	References
*G. lucidum*	Ganoderic acid-β, lucidumol B, ganodermanondiol, ganodermanontriol, and ganolucidic acid A	HIV-1	Inhibits HIV-1 protease	Key for antiretroviral therapy; useful against AIDS	Min et al., 1998
	Ganoderic acid-α, ganoderiol F, and ganodermanontriol	HIV-1	Moderate inhibition of viral replication	May reduce HIV viral load in early stages	El-Mekkawy et al., 1998
	Triterpenoids and polysaccharides	HSV	Blocks viral entry	Potential for cold sore and genital herpes treatment	
	Polysaccharides	Influenza virus	Enhances host immune response	Immunostimulant for seasonal influenza	Li et al., 2015
	Laccases	HIV-1	Inhibits reverse transcriptase	Possible treatment option for resistant HIV strains	Zhang et al., 2011; Flórez-Sampedro et al., 2016
	Polysaccharides	HBV	Inhibits viral replication	May support chronic hepatitis B management	Gao et al., 2003
	Ganoderone C, lucialdehyde B, ergosta-7,22-dien-3α-ol	Influenza virus	Suppresses viral growth	Reduces severity and duration of flu symptoms	Niedermeyer et al., 2005
	Lanosta-trienone (GLTA) and ganoderic acid Y	Enterovirus 71	RNA replication inhibitor	Effective for hand-foot-and-mouth disease in children	Zhang et al., 2014
	Ganoderic acids A–C1, H, and GS-2	HIV	Broad protease inhibition	Potential backbone compounds for HIV therapy	Kang et al., 2015; Cai et al., 2020
	Proteoglycan	HSV-1 and HSV-2	Pre- and co-treatment inhibition	Suitable for both prophylaxis and treatment of herpes	Liu et al., 2014
	Ganoderic acid H	HBV	Suppresses surface antigen expression	Relevant to controlling chronic hepatitis progression	Li and Wang, 2006; Kumar et al., 2020
	Hesperetin, ganosin B	Dengue virus	Inhibits viral protease	Promising approach to limit dengue replication	Lim et al., 2020
*G. adspersum*	Crude extract	HSV-1	Broad-spectrum antiviral activity	Topical applications for recurrent herpes infections	Zahmoul et al., 2024
*G. sinense*	Ganoderiol F, ganoderic acid GS-2, and lucidenic acids	HIV-1	High-affinity viral inhibition	May complement standard HIV therapeutics	El Dine et al., 2008; Sato et al., 2009
*G. colossum*	Farnesyl hydroquinone, ganomycin I and B	HIV-1	Competitive inhibition of protease	Novel anti-HIV leads for drug development	El Dine et al., 2008
*G. lingzhi*	Ganoderic TR and T-Q	H1N1 and H5N1	Neuraminidase inhibition	Potential therapy for influenza pandemics	Zhu et al., 2015
*G. pfeifferi*	Ganodermadiol, lucidadiol, and applanoxidic acid G	Influenza A	Moderate suppression of viral activity	May assist in reducing viral load during flu outbreaks	Mothana et al., 2003

The antiviral effects of *Ganoderma* extend beyond HIV. Polysaccharides and triterpenes from *G. lucidum* have shown inhibitory activity against HSV and influenza virus. These effects are attributed to both direct interference with viral entry and replication, as well as enhancement of host immunity through cytokine stimulation (Basnet et al., 2017). This dual mode of action is particularly relevant in immunocompromised populations where traditional antivirals may fail or cause adverse effects. Notably, *Ganoderma adspersum* extract demonstrated potent activity against HSV-1 with a high selective index and protective efficacy (Zahmoul et al., 2024), highlighting its therapeutic potential for dermatological or mucosal viral infections. Furthermore, triterpenoids such as ganoderiol F, ganodermadiol, and colossolactones isolated from *G. lucidum*, *G. sinense*, and *G. colossum* have shown broad-spectrum activity against HIV-1, HSV, and influenza viruses with IC_50_ or ED_50_ values within pharmacologically relevant ranges (El Dine et al., 2008; Sato et al., 2009; Mothana et al., 2003). These findings suggest that *Ganoderma* may serve as a platform for developing multitarget antivirals—particularly valuable in resource-limited settings where polyvalent therapies are needed to treat co-infections. Although current evidence is largely preclinical, these studies collectively position *Ganoderma*-derived compounds as promising candidates for addressing therapeutic gaps in managing persistent and drug-resistant viral infections. Future efforts should focus on validating these compounds in clinical models and elucidating their pharmacokinetics and immunomodulatory effects to advance their development into viable antiviral therapies.

### 5.4 Parasitic infections

Parasitic diseases continue to exact a significant toll on global health, particularly in tropical and subtropical regions. Malaria alone caused over 600,000 deaths in 2022, predominantly among children under five in sub-Saharan Africa (World Health Organization, 2024). Other parasitic infections, such as toxoplasmosis, giardiasis, leishmaniasis, and blastocystosis, also contribute to considerable morbidity, with limited treatment options, increasing drug resistance, and toxicity issues posing substantial therapeutic challenges. Recent research has highlighted the potential antiparasitic properties of *Ganoderma* species, revealing promising efficacy against several protozoal and parasitic infections (**Table 8**). Notably, nortriterpenes ganoboninketals A–C, derived from *G. boninense* fruiting bodies, demonstrated potent antiplasmodial activity against *Plasmodium falciparum* with IC_50_ values of 4.0, 7.9, and 1.7 μM, respectively (Adams et al., 2010; Ma et al., 2014; **Figure 2**). Additional triterpenes—schisanlactone B, ganodermalactone F, and colossolactone E—isolated from *Ganoderma* sp. KM01 also showed activity against *P. falciparum*, with IC_50_ values ranging from 6.0 to 10.0 μM (Lakornwong et al., 2014). Moreover, *G. lucidum*-derived compounds such as ganoderic acids (DM, TR1, and S), ganodermanondiol, and ganofuran B, isolated using EtOAc, exhibited inhibitory effects on *P. falciparum* within a 6.0–20 μM IC_50_ range (Adams et al., 2010). These activities fall within a biologically relevant range, highlighting their potential as lead compounds for the development of novel antimalarials, especially in the face of rising resistance to artemisinin-based therapies.

**Table 8:** Antiparasitic properties of *Ganoderma* species.

*Ganoderma* species	Active compounds/extracts	Target parasite	Disease relevance/Efficacy	References
*G. boninense*	Ganoboninketals A–C	*Plasmodium falciparum*	Exhibits strong antiplasmodial activity; promising for malaria drug development	Adams et al., 2010; Ma et al., 2014
*G. lucidum*	Ganoderic acids (DM, TR1, and S), ganodermanondiol, and ganofuran B	Targets plasmepsin I enzyme in *Plasmodium*	Inhibits a key enzyme in malaria parasite; potential antimalarial candidates	Adams et al., 2010; Kang et al., 2015
	Hydroalcoholic extract	*Toxoplasma gondii* (RH strain)	More effective than aqueous and alcoholic extracts; potential toxoplasmosis treatment	Ahmadi et al., 2023
*Ganoderma* spp.	Lectins	*Heterodera glycines* and *Ditylenchus dipsaci* (plant-parasitic nematodes)	Limited antiparasitic effect; not viable for agricultural use	Zhao et al., 2009
	Crude extract	*Blastocystis hominis*	Inhibits growth and induces morphological damage; potential for protozoal infection management	Kaewjai et al., 2023; Uwidia et al., 2024
*Ganoderma* sp. KM01	Schisanlactone B, ganodermalactone F, and colossolactone E	*Plasmodium falciparum*	Moderate inhibition; candidates for further antimalarial screening	Lakornwong et al., 2014

In studies on nematode inhibition, Zhao et al. (2009) reported that lectins from *Ganoderma* exhibited activity against plant nematodes *Heterodera glycines* and *Ditylenchus dipsaci*, although their potency was deemed insufficient for practical use. Nonetheless, these findings provide a foundation for future optimization or bioengineering approaches to enhance antihelminthic efficacy. Computational studies further support the antiparasitic potential of *Ganoderma* compounds. *G. lucidum* triterpenoids were shown to interact with plasmepsin I, a key enzyme in *P. falciparum*. Ganodermanondiol demonstrated the highest affinity (binding energy = −7.14 kcal/mol, *K*_i_ = 0.005 mM), outperforming the standard inhibitor KNI-10006 (Kang et al., 2015). This suggests a plausible mechanism of action and reinforces the value of *Ganoderma* constituents in rational drug design against malaria. *Ganoderma* extracts also displayed antiprotozoal effects against *Blastocystis hominis*, a parasite increasingly associated with gastrointestinal disorders. Strong inhibitory activity was observed at an MIC of 62.5 μg/mL. At higher concentrations, extracts of *Ganoderma* and *Boesenbergia rotunda* reduced *B. hominis* growth by up to 90% within 12 h and induced notable morphological damage, pointing to their potential in managing treatment-refractory blastocystosis (Kaewjai et al., 2023; Uwidia et al., 2024). In addition, *G. lucidum* extracts demonstrated anti-*Toxoplasma* effects, particularly against *Toxoplasma gondii* RH strain tachyzoites. *In vitro* studies showed that the hydroalcoholic extract of *G. lucidum* exhibited the highest toxoplasmacidal activity and selectivity (EC_50_ = 3.274), outperforming both aqueous (EC_50_: 76.32) and alcoholic extracts (EC_50_: 40.18) (Ahmadi et al., 2023). Given the limited efficacy and potential teratogenicity of current anti-toxoplasmosis treatments, such natural alternatives may offer safer and more accessible interventions, especially in immunocompromised populations. Overall, these findings suggest that *Ganoderma*-derived metabolites hold considerable promise in addressing parasitic diseases where conventional therapies fall short. Future studies should aim to evaluate their efficacy *in vivo*, explore their mechanisms of action, and assess safety profiles to support clinical translation.

## 6 Clinical studies on antimicrobial properties of *Ganoderma*

*G. lucidum* has been extensively studied for its antimicrobial properties, particularly in laboratory and animal models. *In vitro* studies have demonstrated that its bioactive compounds—mainly polysaccharides and triterpenoids—possess antiviral, antibacterial, and antifungal activities. Despite these promising findings, human clinical evidence remains limited, with most clinical research to date focusing on immune modulation, cancer therapy, and liver protection rather than direct antimicrobial effects. Some preliminary clinical studies suggest potential antiviral benefits. A pilot clinical trial conducted by Hijikata et al. (2005) evaluated an herbal formula containing *G. lucidum* in patients with herpes zoster (shingles). Participants who received 750 mg daily experienced rapid symptom relief, with most resolving within 10 days, and no cases of postherpetic neuralgia were reported after 1 year. In a subsequent study by the same group (Hijikata et al., 2007), individuals with recurrent herpes simplex infections who were treated with a hot water extract of *G. lucidum* at 4 g daily reported faster symptom resolution—genital herpes symptoms improved in 4.9 ± 1.3 days compared to 10.9 ± 6.3 days without treatment. However, both studies involved combination herbal formulas, making it difficult to isolate the specific effects of *G. lucidum*. To date, there are no human clinical trials specifically evaluating the antibacterial efficacy of *G. lucidum*, and evidence in this area is limited to *in vitro* findings. Similarly, while antifungal activity has been reported in laboratory settings—particularly against *Candida* species and dermatophytes—no human studies have validated these effects clinically. Research on its antiparasitic activity remains scarce, with neither significant preclinical nor clinical data currently available.

## 7 *Ganoderma* against plant pathogens

Research on *Ganoderma* has revealed its potential as a natural biocontrol agent against various plant pathogens. Numerous studies have documented its antimicrobial effects, highlighting its capacity to combat fungal and bacterial infections in plants. *G. lucidum* mycelia showed moderate antimicrobial activity against soil-borne pathogens, including fungi (*F. oxysporum*, *Rhizoctonia solani*, and *Sclerotium rolfsii*) and bacteria (*R. solanacearum* and *S. aureus*). *In vitro*, mycelial extracts increased inhibition zones, while *in vivo* tests on tomato seedlings delayed disease symptoms, suggesting *G. lucidum* as a potential biocontrol agent, particularly against *R. solani* and *S. rolfsii* (Mendoza and Nepomuceno, 2006). *G. lucidum* extracts exhibit antifungal properties effective against plant pathogens *F. oxysporum* and *Alternaria alternata* in marigolds. This study compared organic and aqueous extracts of *G. lucidum*, applying various concentrations (5%, 10%, 15%, and 20%) using Agar absorption, Agar well diffusion, and Vapor assay methods. Methanolic extract showed the highest inhibition (64%) using the Agar absorption method, while aqueous extract showed the lowest inhibition (38%) with Agar well diffusion. These findings highlight the potential of *G. lucidum* methanolic extract as a biological control agent for marigold plant diseases (Shahid et al., 2016). The antimicrobial activity of extracts from wood-rotting Basidiomycetes mushrooms from *Eucalyptus* plantations in Uruguay was investigated. Eight extracts, including those from *G. resinaceum* and *L. sulphureus*, were active against pathogens such as *Xanthomonas vesicatoria* and *Aspergillus oryzae* (Barneche et al., 2016). A compound named G_app7, isolated from *G. applanatum*, was found to effectively inhibit the growth of *Sclerospora graminicola*, the pathogen causing downy mildew in pearl millet (*Pennisetum glaucum*). G_app7 reduced sporangium formation (41.4%), zoospore release (77.5%), and motility (91%), and closely resembles metominostrobin, a fungicide. It remained effective at temperatures between 25 and 80°C and was stable for at least 12 months at 4°C. Seed treatment with G_app7 provided a 63% increase in disease protection compared to controls, highlighting its potential as an environmentally safe agrochemical for pearl millet protection (Jogaiah et al., 2016).

The antibacterial effects of selenium-containing biocomposites from submerged cultures of *Ganoderma* species were studied against plant pathogenic bacteria. Biocomposites from *G. cattienensis* and *G. lucidum* were most effective against *C. michiganensis*, while those from *G. valesiacum* and *G. lucidum* showed strong activity against *Xanthomonas campestris*. *G. colossus* exhibited notable activity against *Pseudomonas fluorescens*. The study highlights the potential of using coumarin-based compounds for producing antimicrobial substances from fungi (Perfileva et al., 2017). Eight mushroom species were screened, including *G. lucidum*, for their impact on *Colletotrichum capsici*, the chili fruit rot pathogen. The results revealed that *G. lucidum*, *Auricularia polytricha*, and *Lentinus edodes* demonstrated significant antifungal activity, with *G. lucidum* achieving the highest mycelial growth inhibition (54.81%). Chloroform extracts from *G. lucidum* inhibited spore germination (88%) and mycelial growth (60.55%) at 24 h. These findings suggest *G. lucidum* as a promising source for developing fungicides against *C. capsici*, warranting further investigation of its active compounds (Priy et al., 2019).

The antimicrobial potential of an aqueous ammonia extract from *G. lucidum* carpophores, sourced from *Quercus ilex* trees, was investigated, revealing key chemical constituents such as acetamide and oleic acid. The extract exhibited strong anti-oomycete and antifungal activities, with MIC values of 187.5 μg·mL^−1^ against *Phytophthora cinnamomi* and varying MICs against other fungi. When conjugated with chitosan oligomers, the extract’s antimicrobial efficacy significantly increased, showcasing MIC values as low as 78.12 μg·mL^−1^, demonstrating its potential for protecting holm oak in sustainable agricultural practices (Sánchez-Hernández et al., 2023). The antifungal properties of *G. lucidum* against the mango anthracnose pathogen *C. gloeosporioides* were investigated in this study. Ethyl acetate extracts from the fruiting body inhibited mycelial growth by 70.10% at a 1% concentration. Thin-layer chromatography identified two active bands, with the first achieving 53.77% inhibition. Gas chromatography–mass spectrometry detected benzothiazole, which completely inhibited mycelial growth at 50 ppm and caused structural abnormalities in the pathogen. The findings suggest that *G. lucidum* biomolecules could be effective natural agents against plant pathogens (Muniyappan et al., 2023). The crude extract of *G. lucidum* was formulated into an emulsion [water in oil (W/O)] to induce systemic resistance in chickpeas against *Fusarium* wilt caused by *F. oxysporum* f. sp. *ciceri* (FOC). Different dilutions of the formulation were applied to chickpeas, which were then challenged with FOC. Enzyme assays showed increased activity of peroxidase (PO), polyphenol oxidase (PPO), and phenylalanine ammonia-lyase (PAL) in treated plants, indicating activation of the plant’s natural defense pathways. GC-MS analysis confirmed bioactive compounds responsible for enhancing enzyme levels. This study suggests the potential for developing bio-formulations to control plant diseases (Singh and Vyas, 2023). **Table 9** summarizes the antimicrobial activities of *Ganoderma* species against plant pathogens.

**Table 9:** Antimicrobial activity of *Ganoderma* spp. against plant pathogens.

*Ganoderma* species	Target pathogen(s)	Type of activity	Key findings	Disease relevance	References
*G. applanatum*	*Sclerospora graminicola* (pearl millet downy mildew)	Antifungal *(in vitro*)	Isolate G_app7 suppressed spore formation and improved plant resistance	Potential bioagent for downy mildew control in cereals	Jogaiah et al., 2016
*G. cattienensis* and *G. lucidum*	*Clavibacter michiganensis*, *X. campestris*, and *P. fluorescens*	Antibacterial (selenium biocomposites)	Selenium nanoparticles from Ganoderma selectively inhibited bacterial growth	Useful for agricultural pathogen control and seed coating	Perfileva et al., 2017
*G. lucidum*	*Phytophthora cinnamomi* and other phytopathogens	Antifungal and anti-oomycete	Efficacy enhanced by chitosan combination	Effective against root rot and damping-off diseases	Sánchez-Hernández et al., 2023
	*Fusarium oxysporum* f. sp. *ciceri* (chickpea wilt)	Induced systemic resistance	Stimulated plant defense enzymes (PO, PPO, and PAL)	Sustainable control of *Fusarium* wilt in legumes	Singh and Vyas, 2023
	*Colletotrichum gloeosporioides* (mango anthracnose)	Antifungal (*in vitro*)	70% inhibition of mycelial growth; benzothiazole identified	Potential for pre-harvest mango protection	Muniyappan et al., 2023
	*F. oxysporum*, *R. solani*, *S. rolfsii*, and *R. solanacearum*	Antifungal, antibacterial (*in vitro* and *in vivo*)	Mycelial extract delayed disease onset and increased inhibition zones	Broad-spectrum plant disease control	Mendoza and Nepomuceno, 2006
	*F. oxysporum* and *Alternaria alternata* (marigold pathogens)	Antifungal (*in vitro*)	Methanolic extract had 64% growth inhibition	Alternative to chemical fungicides for ornamentals	Shahid et al., 2016
	*Colletotrichum capsici* (chili fruit rot)	Antifungal (*in vitro*)	Inhibited spore germination (88%) and mycelial growth (54.8%)	Biocontrol option for chili postharvest spoilage	Priy et al., 2019
*G. resinaceum* and *Laetiporus sulphureus*	*X. vesicatoria* and *Aspergillus oryzae*	Antibacterial and antifungal (*in vitro*)	Crude extracts suppressed growth of pathogens from *Eucalyptus* plantations	Supports integrated pest management in forestry	Barneche et al., 2016

## 8 Challenges and limitations of *Ganoderma* in antimicrobial applications

Although *Ganoderma*, especially *G. lucidum*, has demonstrated promising antimicrobial properties, several key challenges limit its broader adoption in medical and agricultural settings. These challenges primarily stem from variability in species, inconsistency in extract composition, and a lack of robust human clinical research specifically targeting antimicrobial use. A major hurdle is the natural variation in bioactive compounds among different *Ganoderma* species. Each species produces a unique blend of compounds—such as polysaccharides, triterpenoids, and phenolics—which directly influences their antimicrobial efficacy. Even within the same species, factors like geographical origin, climate, substrate, and cultivation conditions can alter the concentration and types of active molecules. This variability makes it difficult to predict or compare the antimicrobial strength of different extracts, reducing their reliability as standardized treatments. Another significant limitation lies in the difficulty of standardizing *Ganoderma* extracts. Unlike conventional pharmaceuticals that are based on single, well-defined molecules, *Ganoderma* extracts are complex mixtures. Depending on the extraction method used—whether water-based or alcohol-based—the resulting compounds and their concentrations can vary greatly. This leads to inconsistent therapeutic profiles, making dosage optimization and reproducibility a challenge. Furthermore, there is currently no universally accepted quality control standard for *Ganoderma* products, which adds another layer of uncertainty for clinical or commercial use. Perhaps the most critical limitation is the lack of extensive human clinical trials specifically designed to assess antimicrobial effects of *Ganoderma*. While laboratory and animal studies have shown promising results against bacteria, fungi, and viruses, human trials remain scarce. Most clinical research has focused on immune modulation, cancer support, and liver protection, rather than on infectious diseases. Without rigorous clinical testing, questions remain about its safety, appropriate dosing, and real-world efficacy. This lack of data also presents a barrier to regulatory approval and mainstream medical acceptance, hindering the development of *Ganoderma*-based antimicrobial therapies. Although *Ganoderma* holds great promise as a natural antimicrobial agent, issues related to species variability, extract standardization, and insufficient clinical evidence must be addressed before it can be reliably integrated into therapeutic or agricultural practices.

## 9 Future research directions for *Ganoderma* in antimicrobial applications

The growing recognition of antimicrobial properties of *Ganoderma* highlights several critical research avenues that could unlock its full therapeutic potential. First and foremost, standardizing *Ganoderma* extracts is essential to ensure consistency in their bioactive compounds, such as polysaccharides, triterpenoids, and phenolics. Variations in species, cultivation methods, and extraction techniques currently lead to unpredictable antimicrobial effects, limiting reproducibility in both research and clinical applications. Future studies should focus on optimizing extraction protocols and determining minimum effective concentrations to create reliable, high-quality formulations suitable for pharmaceutical use. Another promising direction involves developing *Ganoderma*-based antimicrobial drugs or supplements. Its bioactive compounds have demonstrated broad-spectrum activity against bacteria, fungi, and viruses, making them strong candidates for novel treatments. Given the escalating threat of AMR, *Ganoderma*’s multi-target mechanisms—including cell wall disruption, nucleic acid synthesis inhibition, and oxidative stress induction—could provide alternative therapies that pathogens struggle to resist. Perhaps most compelling is the potential for *Ganoderma* to enhance conventional antibiotics through synergistic combinations. Preliminary evidence suggests that pairing *Ganoderma* extracts with existing antimicrobials may improve efficacy while reducing required dosages, thereby minimizing side effects and delaying resistance. Future research should systematically investigate these interactions, particularly against drug-resistant strains, as well as explore the role of *Ganoderma* as an adjunct therapy for fungal and viral infections in immunocompromised patients. By addressing these priorities, *Ganoderma* could transition from a traditional remedy to a scientifically validated antimicrobial agent, offering new solutions in an era of increasing treatment challenges.

## 10 Conclusion

*Ganoderma* exhibits significant promise as a natural source of antimicrobial agents, with its bioactive compounds—polysaccharides, triterpenoids, phenolic compounds, and proteins—demonstrating a variety of mechanisms to combat bacterial, fungal, and viral infections. These compounds function by disrupting microbial cell walls, inhibiting nucleic acid synthesis, modulating the immune system, and regulating oxidative stress, offering a multi-targeted approach to pathogen inhibition. However, it is important to note that there may be potential risks or limitations associated with the use of *Ganoderma* as an antimicrobial agent, which should be thoroughly investigated in future research. Numerous *in vitro* and preclinical studies have already illustrated *Ganoderma*’s potential to be developed into therapeutic agents, especially in light of the growing global concern over AMR. Future research should prioritize clinical trials to validate *Ganoderma*’s efficacy in human subjects, particularly for its antimicrobial applications. Standardizing *Ganoderma* extracts is another critical area that would facilitate consistency in research and therapeutic use. In addition, identifying and isolating specific active compounds within *Ganoderma* may allow for more targeted drug development, potentially leading to the creation of new antimicrobial drugs or supplements. Furthermore, exploring synergistic effects with conventional antibiotics could offer new solutions to enhance treatment efficacy and reduce drug resistance. Continued investigation into these areas will be key to unlocking *Ganoderma*’s full potential as a vital player in the future of antimicrobial therapies.
